# Marine primary production enhances removal of hydrophobic organic contaminants from Eurasian Arctic shelf waters

**DOI:** 10.1038/s41467-026-78857-3

**Published:** 2026-10-23

**Authors:** Xiaodi Shi, Laurent Oziel, Jonathan P. Benskin, Örjan Gustafsson, Anna Sobek

**Affiliations:** 1 Department of Environmental Science, Stockholm University, Stockholm, Sweden; 2 Alfred-Wegener-Institut - Helmholtz-Zentrum für Polar- und Meeresforschung, Bremerhaven, Germany; 3 CNRM, Université de Toulouse, Météo-France, CNRS, Toulouse, France; 4 The Bolin Centre for Climate Research, Stockholm University, Stockholm, Sweden

**Keywords:** Environmental monitoring, Carbon cycle

## Abstract

Global oceans store large quantities of human-made persistent hydrophobic organic contaminants (HOCs), driven by the organic carbon (OC) cycle. Despite diverse organic matter composition, bulk OC is typically treated as a uniform sorbent when assessing HOC fate. Significant uncertainty remains regarding the factors controlling variability in the sorption of HOCs by organic matter. Using surface sediments across the Eurasian Arctic shelves, spanning a land-to-ocean continuum of OC, we show that polychlorinated biphenyl concentrations per unit marine‑derived OC are up to an order of magnitude higher than those per unit terrestrial OC. Coupling these empirical relationships with a state‑of‑the‑art ocean biogeochemistry model shows that climate‑driven increases in marine primary production in the Eurasian Arctic shelves lead to 1.3 times more HOC removal from the water column. These findings reveal that both OC quantity and quality control the sediment HOC sink and that climate‑induced shifts in OC cycles may restructure HOC fate in the Arctic Ocean.

## Abstract (web summary)

Measurements of hydrophobic pollutants and organic matter in sediments across the Eurasian Arctic show that sediments dominated by marine organic matter are more efficient sinks for hydrophobic pollutants than terrestrially dominated sediments.

## Introduction

Human-made persistent contaminants have dispersed globally, reaching the remote Arctic and deep hadal trenches^1,2^. Global oceans receive persistent contaminants through atmospheric transport and deposition, riverine discharge, and ocean currents. Among these persistent contaminants, hydrophobic compounds preferentially concentrate in organic matter through multimedia partitioning and subsequently sink to sediments and deeper waters^3,4^. The tight coupling between the marine organic carbon (OC) cycle and the fate of hydrophobic organic contaminants (HOCs) drives storage of large quantities of HOCs in sediments and deep waters^3–6^, e.g., accounting for approximately 60% of total polychlorinated biphenyl (PCB) emissions^7^. When modeling the fate of HOCs, bulk OC is often treated as a chemically uniform sorbent^8–10^, despite evidence that its sorption potential can vary by up to three orders of magnitude^11,12^.

The variability in sorption potential of organic matter can be largely attributed to its diverse chemical composition, driven to a large extent by its origin and degradation^13–15^. Terrestrial organic matter mainly consists of plant litter and highly decomposed soil humus, whereas marine organic matter carries a greater fraction of lipid‑rich phytoplankton residues^13,14^. Aromaticity as found in carbon structures of terrestrial organic matter is beneficial for sorption of aromatic and halogenated contaminants^16–18^, through increasing polarizability and forming charge-transfer complexes (π−π interactions)^12^. However, a sorption experiment with a variety of organic matter samples, including humic acid extracts and algae, showed that sorption potential is positively associated with aliphatic carbon content (more present in marine organic matter), while no associations were found between sorption potential and aromatic carbon content^19^. Laboratory measurements of sorption potential of soil and sediment samples from widely diverse geographic regions revealed that the sorption potential of marine sediment organic matter from bays/harbors can be two times higher than that of soil organic matter for HOCs^20,21^. Existing data from sorption studies on phytoplankton and soil suggest that phytoplankton sorption potential is about three times higher than that of soil organic matter^22–25^. The only field‑based support from a study with quantified fractions of marine OC (*f*_mar_) in sediment likewise showed that sorption of HOCs in the Baltic Sea was 2–10 times higher at the site with higher *f*_mar_ (89%) compared to lower *f*_mar_ (60%)^26^. However, *f*_mar_ in sediments spans from 33 ± 24% in deltas to >90% in the open sea^13,26,27^, requiring insights into the impacts of organic matter on HOC fate throughout the land-to-ocean continuum. An empirical and quantitative assessment across larger spatial gradients of sediment *f*_mar_ would help improve our understanding of how OC origin influences HOC sorption and mobility in the global oceans.

**Fig. 1:**
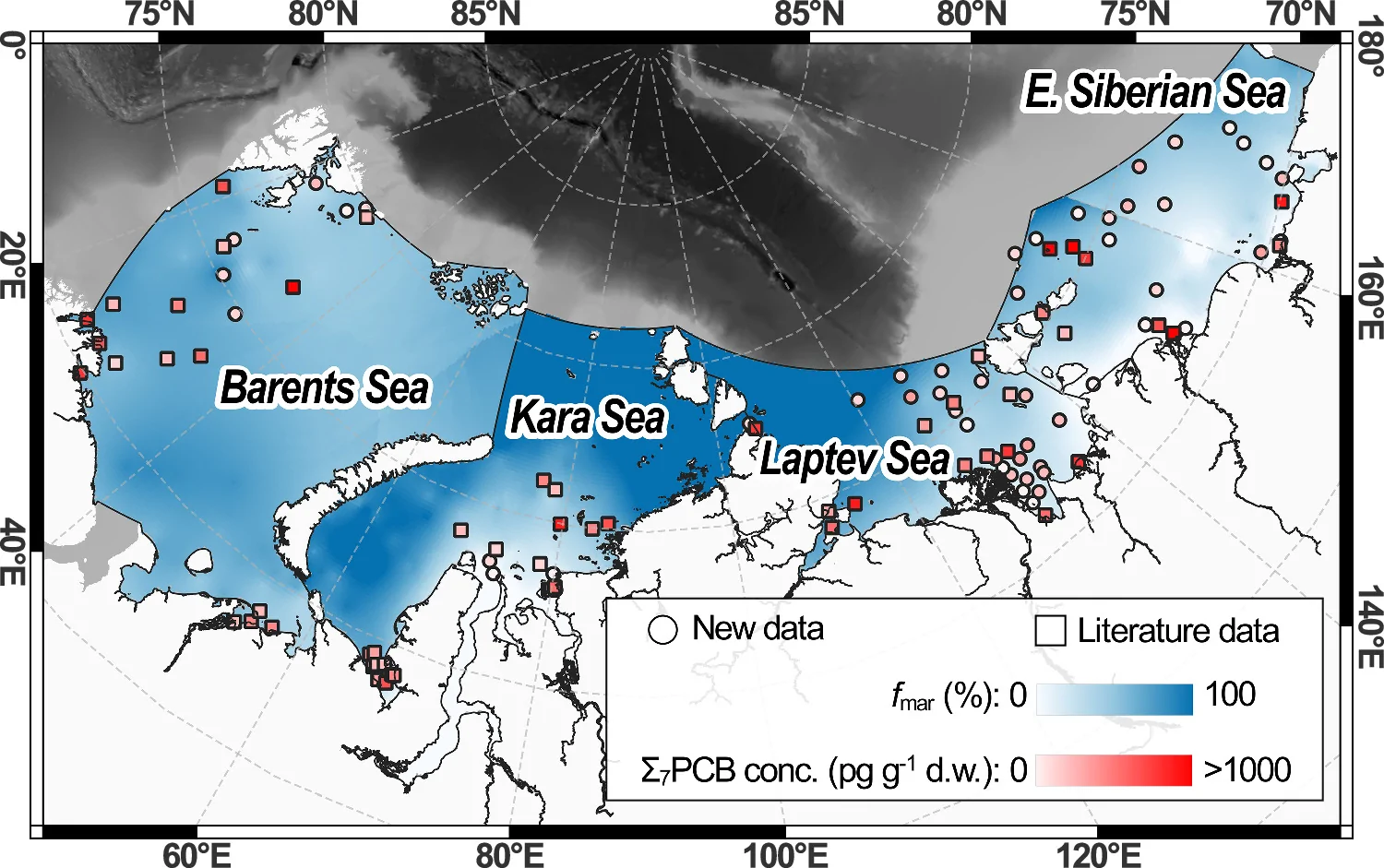
The land-to-ocean continuum of organic carbon (OC) and dry weight (d.w.)-based concentrations of polychlorinated biphenyls (PCBs) across the Eurasian Arctic shelves. The fraction of marine OC (*f*_mar_) in surface sediments was derived from interpolated δ^13^C values from the Circum‑Arctic Sediment Carbon DatabasE (CASCADE; see Methods regarding calculation of *f*_mar_ in sediment)^30^. The full land-to-ocean continuum of OC enables quantitative assessment of the influence of OC origin on the sediment sink of hydrophobic organic contaminants. Base map from IBCAO v4^77^; sea boundaries from the International Hydrographic Organization^78^. Σ_7_PCB: Sum of polychlorinated biphenyl #28, #52, #101, #118, #138, #153, and #180. The figure was created using QGIS (version 3.40.8).

The Eurasian Arctic shelves provide an ideal and unique natural laboratory for examining the influence of the land-to-ocean continuum of OC sorbent properties on HOC sinks. The Arctic Ocean accounts for 2.5% of the global ocean area but 7-11% of global OC burial^28^. Particularly, the Eurasian Arctic shelves receive roughly five times more terrestrial OC from coastal erosion and riverine runoff than the American Arctic^28,29^. The recently developed Circum‑Arctic Sediment Carbon Database (CASCADE), comprising more than 4000 total OC and over 1500 δ^13^C data points in surface sediments, enables the estimation of *f*_mar_ across the region (Fig. 1)^30^. Rapid Arctic warming, about three times the global average^31^, is potentially altering the OC cycle of the Arctic Ocean^32^, which could exert substantial influence on the HOC sink in the Arctic^8^.

Here, we test whether marine organic matter enhances HOC accumulation in sediments and examine how climate‑driven changes in the OC cycle modulate the HOC sink on the Eurasian Arctic shelves. We use seven indicator PCBs (#28, #52, #101, #118, #138, #153, and #180) as model HOCs, because of their extensive historical production, strong coupling to biogeochemical processes following their global phase‑out, wide range of hydrophobicity, and extensive quantification in the Arctic^4,33–36^. We compile newly generated and previously reported congener‑specific PCB concentrations in surface sediments from 111 sites across the Eurasian Arctic shelves (Fig. 1; Tables S1-2 in the Supplementary Dataset) to evaluate OC‑normalized contaminant concentrations as a function of δ^13^C‑derived *f*_mar_ (Tables S1-2 in the Supplementary Dataset). Coupling these empirical PCB concentrations in OC with the state‑of‑the‑art ocean–ice biogeochemistry model FESOM2.1‑REcoM3^37^ enables us to further estimate variations in the Arctic shelf HOC sink over the 21^st^ century. Our findings suggest that PCB concentrations in sediment dominated by marine OC are an order of magnitude higher than those in terrestrially-dominated sediment OC and, consequently, that climate‑driven shifts in quantity and, importantly, also quality of organic matter may restructure the HOC sink in the Arctic shelves.

## Results and discussion

### Consistent PCB patterns in Eurasian Arctic shelf sediments

To capture PCB concentration variability across the Eurasian Arctic shelves, we measured individual PCB concentrations in surface sediments from 52 sites (Fig. 1). To fill spatial gaps, particularly in the Barents and Kara Seas, we retrieved previous observations of seven indicator PCBs from 47 sites reported in the Arctic Monitoring and Assessment Program (AMAP) assessment report (1998), as well as from 12 additional sites obtained from two databases (i.e., MarChem: The Marine Chemistry database for Norwegian waters and ICES: International Council for the Exploration of the Sea; Fig. 1)^38–40^. Sampling information, biogeochemical parameters, and PCB concentrations for the new measurements are summarized in Table S1 (Supplementary Dataset) and literature data in Table S2 (Supplementary Dataset).

We evaluated the comparability and variability of this combined dataset to ensure that spatial patterns reflect biogeochemical controls rather than data artefacts. Most sum concentrations of seven indicator PCBs (Σ_7_PCB concentrations) fall below 1 ng g^−1^ dry weight, which is consistent with values reported for other Arctic Ocean regions (see Table S3 in Supplementary Information for a summary of previous PCB measurements in Arctic surface sediments). Σ_7_PCB concentrations within each shelf sea significantly declined by 50-60% between 1994 and 2020, in line with observed long-term declines in PCB air concentrations in the Arctic following their production phase‑out^41^. Measured congener profiles in surface sediments align well with previous measurements in the water column for each shelf (see Figure S1 in Supplementary Information for congener distribution)^42^. PCB28 dominates congener profiles, with its contribution decreasing from east to west (61% in the East Siberian Sea to 19% in the Barents Sea), indicative of atmospheric delivery of more volatile congeners^39^. Contributions of hexa‑ and hepta-PCBs increase westward (from 15% in the East Siberian Sea to 37% in the Barents Sea), reflecting the proximity of western shelves to former high‑emission regions in northeastern North America and northern Europe^43^. Overall, these expected temporal and longitudinal patterns support the reliability of the assembled database. Additional quality assessments are provided in Section B in Supplementary Information.

We further examined latitudinal trends for individual PCB congeners using subsets of samples with comparable sampling years (i.e., sampling year within six years). Neither dry‑weight nor OC‑normalized PCB concentrations show significant decreases between 70°N and 80°N. In the Laptev Sea, sediment concentrations of PCB118, 138, and 153 even significantly increase northward. Long-range transport of PCBs occurs by global fractionation driven by temperature and physicochemical properties of the PCB congeners. For example, decreasing temperature northwards results in condensation of PCBs out of the gaseous phase and thus less latitudinal transport^39^. Decreases of northward transport are steeper for less volatile PCBs^39^. Previous measurements have shown decreasing PCB transport toward the high Arctic, such as a two‑fold decline in PCB seawater concentrations from 71°N to 80°N along a transect from the Norwegian Sea to the North Pole in 2001, as well as a two‑fold decrease in seawater concentrations and a seven‑fold decrease in atmospheric concentrations from 68°N to 80°N in the Greenland Current and the Atlantic sector of the Arctic Ocean in 2007^44,45^. The stable, even increasing, latitudinal trends in sediment concentrations observed in this study therefore suggest that other factors decouple the PCB sink from declining northward PCB transport. δ^13^C-drived *f*_mar_ in sediments increases significantly toward higher latitudes, e.g., from 10% to 97% in the Laptev Sea^29^. Previous sorption experiments and limited field observations have reported at least two‑fold higher sorption of HOCs to marine organic matter relative to terrestrial organic matter^20,21,26^. We therefore hypothesize that increasing *f*_mar_ could be one of the factors that offset the expected latitudinal decline in PCB transport, resulting in the spatially uniform PCB concentrations observed across the Eurasian Arctic shelf sediments.

### Enhancement effect of marine organic matter on PCB sediment concentrations

To quantify the influence of *f*_mar_ on PCB concentrations in Arctic shelf sediments, we first attempted to disentangle any effects of other major factors controlling PCB concentrations, namely region, sampling year, and latitudinal decline in PCB transport. Sites were classified into eastern (i.e., Laptev and East Siberian Seas) and western (i.e., Barents and Kara Seas) regimes, based on their congener profiles (Fig. S1 in Supplementary Information). We corrected for temporal variations and latitudinal transport using long‑term PCB trends in atmospheric concentrations measured at the Zeppelin station, Svalbard (79° N, 12° E) from 1993 to 2012 and surface seawater concentration gradients from the Norwegian Sea to the central Arctic in 2001 (see Methods for details of correction), respectively^41,44^. The resulting OC‑normalized PCB concentrations, standardized to a latitude of 75°N and a depositional year of 2008, are listed in Tables S1-2 (Supplementary Dataset).

**Fig. 2:**
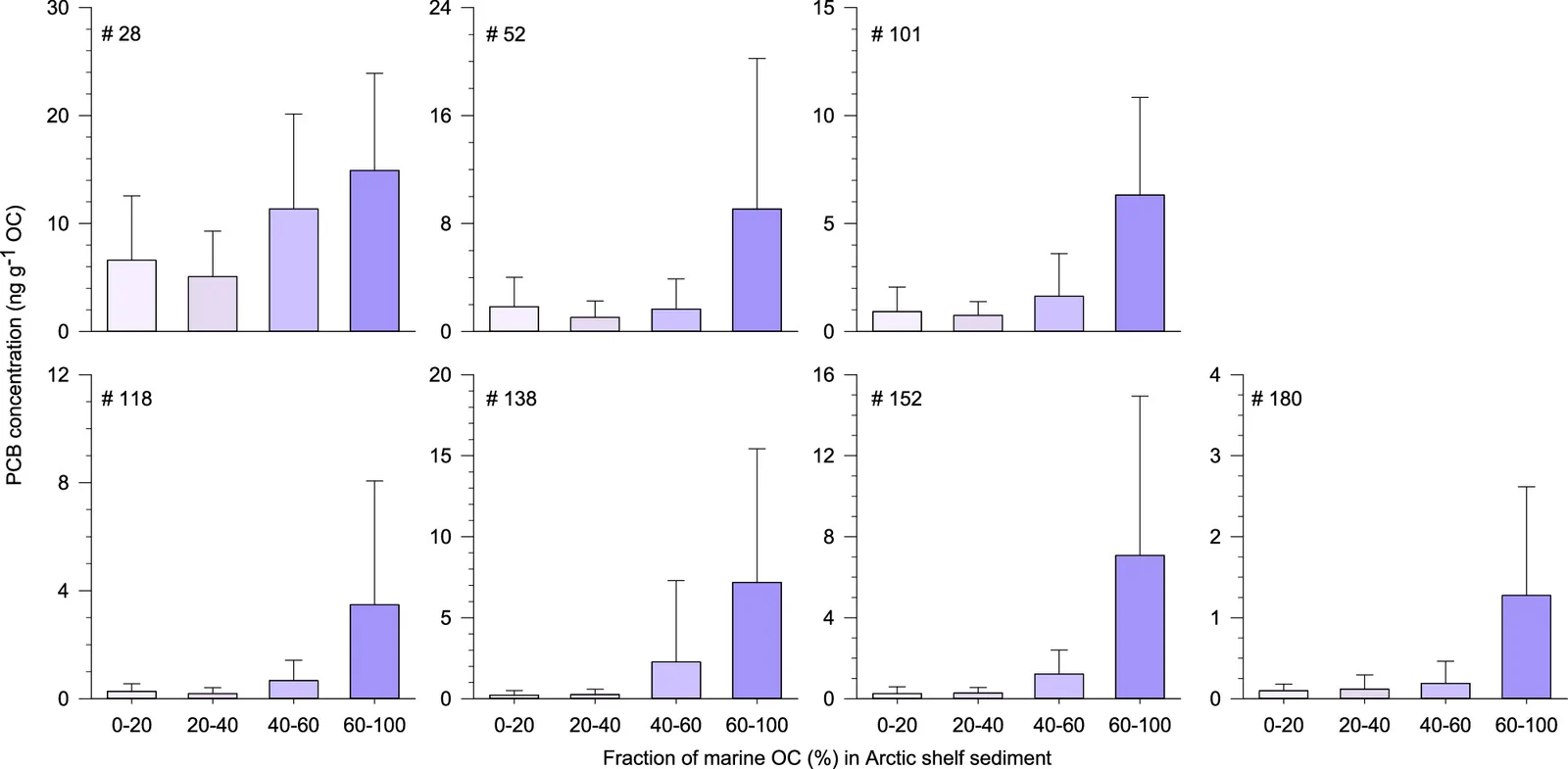
Enhanced organic carbon (OC)‑normalized polychlorinated biphenyl (PCB) concentrations (ng g^-1^ OC) in marine OC‑dominated sediments. OC‑normalized concentrations in surface sediments from the Laptev and East Siberian Seas, after correction for effects of latitudinal transport (standardized to 75°N) and time (standardized to 2008) on PCB sediment concentrations. Bars represent mean values and whiskers indicate standard deviations (*n* = 64). Colors from bright to dark indicate fraction of marine OC from low to high.

OC‑normalized concentrations of individual PCBs were higher at sites dominated by marine OC (*f*_mar_: 64-100 and 62-73% in the eastern and western regimes, respectively) than at sites dominated by terrestrial OC (*f*_mar_: 0-16 and 0-24 %). In the eastern regime, PCB concentrations were approximately twice as high for tri‑chlorinated PCBs at high-*f*_mar_ sites, 5 times for tetra‑chlorinated PCBs, 7-11 times for penta-chlorinated PCBs, and 20-30 times for hexa-chlorinated PCBs (Fig. 2). In the western regime, concentrations of penta‑, hexa‑, and hepta-chlorinated PCBs were significantly higher at high-*f*_mar_ sites, whereas differences for other congeners were not significant (Fig. S2 in Supplementary Information). The suggested enhancement effect of marine organic matter on PCB sediment concentrations increased with PCB hydrophobicity. A previous comparison of site‑specific organic carbon–water partition coefficients (*K*_oc_) between two sites with different *f*_mar_ in sediment from the Baltic Sea likewise showed that *K*_oc_ differences were more pronounced for the most hydrophobic PCBs^26^. These findings agree with the thermodynamic partitioning mechanism for HOC sorption, in which sorption potential exerts stronger control on more hydrophobic compounds.

**Fig. 3:**
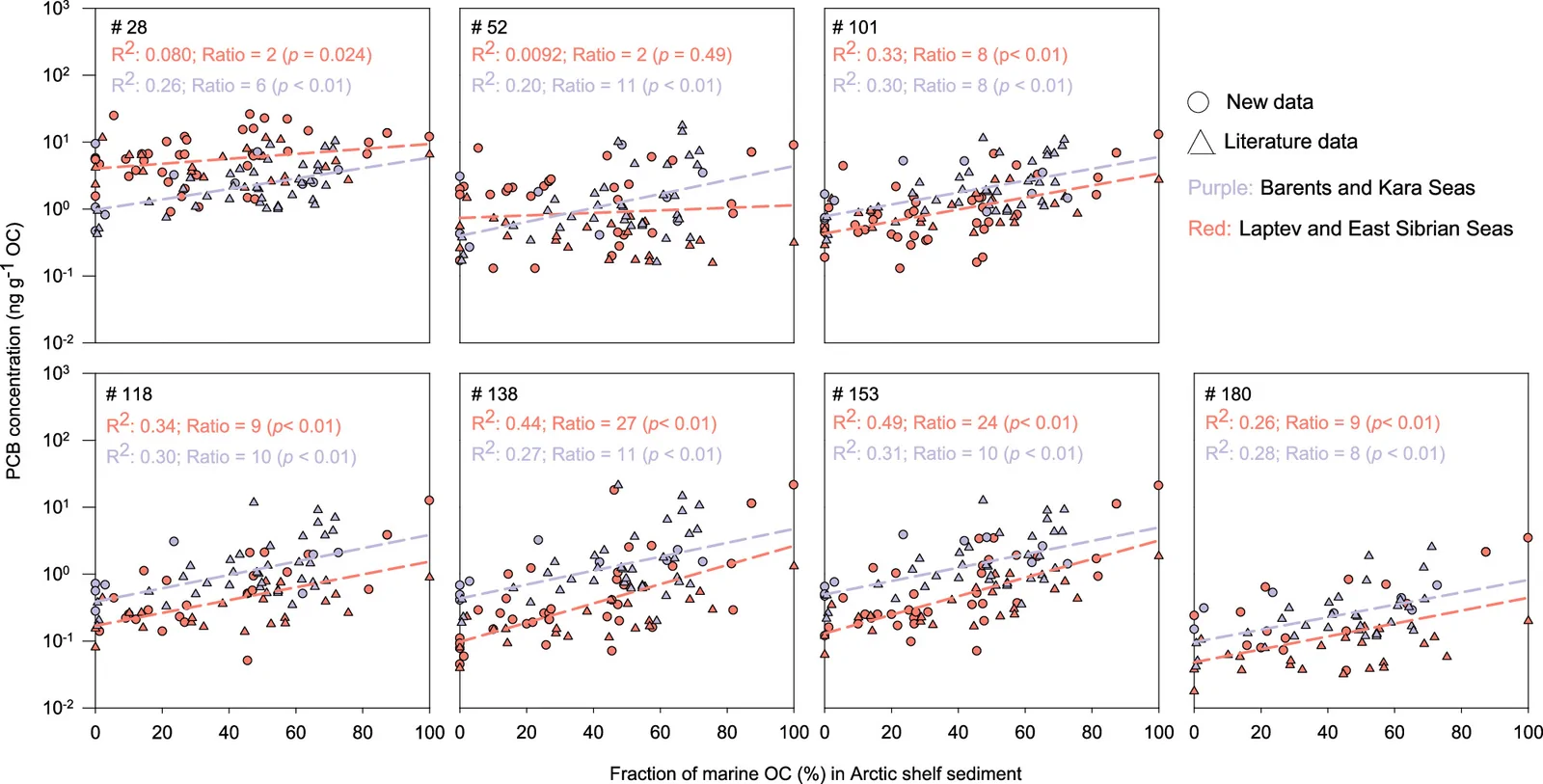
Organic carbon (OC) origin controls sorption of polychlorinated biphenyls (PCB) across the land-to-ocean continuum of OC. Relationships between the δ¹³C‑derived fraction of marine OC (%) and corrected OC‑normalized concentrations (ng g⁻¹ OC) of seven PCB congeners in surface Arctic shelf sediments. Data are standardized to a latitude of 75°N and a depositional year of 2008. Red and purple symbols denote eastern (Laptev and East Siberian Seas) and western (Barents and Kara Seas) regimes, respectively. Circles and triangles indicate new and literature data, respectively. Regression‑based extrapolations indicate up to one order‑of‑magnitude higher PCB concentrations per unit marine OC relative to those per unit terrestrial OC.

We established regressions between δ^13^C-derived *f*_mar_ and standardized OC‑normalized concentrations in sediment to estimate differences in sorption potential between marine and terrestrial organic matter (Fig. 3; see Table S4 in Supplementary Information for a summary of regression results). Extrapolated PCB concentrations per unit marine OC (ng g^-1^ OC; *f*_mar_ = 100%) were on average one order of magnitude higher (6−11 times) than those per unit terrestrial OC (*f*_mar_ = 0%) in the western regime. The mean enhancement of PCB concentration between marine and terrestrial OC in the eastern regime was 13-fold, with nearly 27-fold maxima for hexa-chlorinated PCBs. An order-of-magnitude difference in PCB concentration between marine and terrestrial OC indicates that quantity and, importantly, also quality of organic matter are critical for HOC fate and bioavailability.

Further climate‑driven changes have the potential to alter the quantity and origin of organic matter through changes in marine primary production, coastal erosion, and riverine inputs^46,47^. As a consequence, vertical transport and air‑to‑water fluxes of HOCs may be affected, thereby reshaping the residence times and bioavailability of HOCs in marine systems.

The present study provides large‑scale and quantitative support for the enhancement effect of marine organic matter on the HOC sediment sink, based on observations from 111 sites spanning the full land-to-ocean continuum of OC across the Eurasian Arctic shelves. Several previous field studies have suggested similar effects, although without quantitative characterization of OC origin^48–51^. Sediments from the eastern Indian Ocean exhibited higher PCB concentrations in samples with lower ratios of total organic carbon to total nitrogen (TOC/TN), suggesting stronger sorption to marine organic matter^48^. In the Baltic Sea, offshore sediments with higher marine organic matter influence contained higher OC-normalized PCB concentrations than terrestrially influenced coastal sediments^49,50^. Large‑scale assessments across the Baltic Sea further indicated elevated sediment-water PCB distribution coefficients in regions with high phytoplankton‑driven organic matter deposition^51^. Only one field study compared *K*_oc_ at two sites with quantified *f*_mar_ in sediment (60 and 89%) in the Gulf of Finland, Baltic Sea, reporting 5-9‑fold *K*_oc_ differences for penta‑ to hepta‑chlorinated PCBs^26^. Considering the small difference in *f*_mar_ in sediment between these two sites, the enhancement effect of marine organic matter on the HOC sink is comparable to the current estimation.

A mechanistic explanation for higher HOC concentrations in marine compared to terrestrial organic matter likely lies in the abundance of aliphatic carbon derived from phytoplankton debris^13^, which exhibits strong affinity toward HOCs^12,19,52^. Sorption experiments showed that phenanthrene *K*_oc_ increased with aliphatic carbon content, with degraded algal matter and collagen exhibiting the highest values and cellulose and lignin, two major soil components, the lowest^19^. A comprehensive compilation of sorption data likewise demonstrated the strong affinity of HOCs for aliphatic organic matter and proposed that its mobile amorphous sorption domain facilitates dissolution of HOCs into organic matter^12^. Additionally, lipids are less polar than humic acid, a major component in soil^53^. Therefore, the negative association between the paraffinic carbon content and polarity further increases the sorption potential of organic matter for HOCs^12,54^.

Although PCB concentrations per unit OC are positively associated with *f*_mar_ in sediment, variability in OC-normalized PCB concentrations remains considerable (Fig. 3). The lack of porewater PCB concentrations from the Eurasian Arctic shelves prevents direct *K*_oc_ calculations, and the temporal and latitudinal corrections introduce uncertainties. Consequently, the mean R^2^ for the statistically significant regressions between OC-normalized PCB concentrations and *f*_mar_ in sediment is 0.30 (correlation coefficient of 0.55; Fig. 3), indicating that OC origin alone cannot explain all variability in sediment storage of HOCs. One source of variability may be the heterogeneity within marine organic matter. For instance, sediments from eutrophic regions of Hudson Bay contain elevated PCB concentrations and higher proportions of hydrophobic congeners relative to oligotrophic regions^55^. Oligotrophic organic matter is likely weathered during repeated cycles of deposition and resuspension, while eutrophic organic matter is freshly generated and potentially a better sorbent to HOCs^55^. Earlier studies in the Baltic Sea and the Svalbard archipelago demonstrated that PCB partitioning to phytoplankton can vary with community composition^45,56^. Additionally, this study uses sediment samples to infer sorption of HOCs to organic matter in the water column. Organic matter sorption and degradation processes in the water column can contribute to variability of HOC concentrations in the sediment. For instance, earlier field studies showed that sorption of HOCs to phytoplankton was negatively associated with biomass in the water phase^57,58^, and degradation of organic matter during vertical transport could amplify HOC concentrations per remaining organic matter^59^. Marine organic matter is preferentially degraded relative to terrestrial organic matter during vertical transport^13^. Nevertheless, we observe significant associations between OC-normalized PCB concentrations and *f*_mar_ in sediment, even for sites from multiple studies across large spatial gradients with high variability. This result highlights the high influence of OC origin on the HOC sink in Arctic shelf sediments.

### Climate-driven alteration of the HOC sink in Eurasian Arctic sediments

To assess how the magnitude of the HOC sediment sink responds to climate‑driven shifts in OC dynamics on the Arctic shelves, we estimated marine OC export fluxes to the sediment bed under a plausible high-emission scenario (Shared Socioeconomic Pathway SSP 3-7.0) and a low-emission scenario (SSP 1-2.6) using the global ocean and sea‑ice biogeochemistry model FESOM2.1‑REcoM3^37,60^. Those high-resolution (4 km) simulations consider varying carbon and nutrient inputs from rivers and coastal erosion. We further estimated PCB exports using extrapolated PCB concentrations in marine OC (Fig. 3). Time‑series data of annual marine OC export fluxes and the PCB sink changes associated with marine OC between two scenarios are provided in Table S5 (Supplementary Dataset). Please see Methods regarding limitations and uncertainties of these estimations.

The annual mean marine OC export fluxes to the sediments of the Eurasian Arctic shelves are 30 Tg year^−1^ under both emission scenarios. These fluxes are about three-fold greater than the annual total OC input from coastal erosion (~9 Tg year^−1^) in the same region and roughly 20-fold for particulate OC supplied by the five major Arctic rivers (~1.6 Tg year^−1^; Ob, Yenisey, Lena, Indigirka, and Kolyma Rivers)^28,61^. In combination with the one‑order‑of‑magnitude higher sorption potential of marine compared to terrestrial organic matter, these results reveal the dominant control of marine primary production on the HOC sink in the Eurasian Arctic shelves through the generation and export of organic matter.

**Fig. 4:**
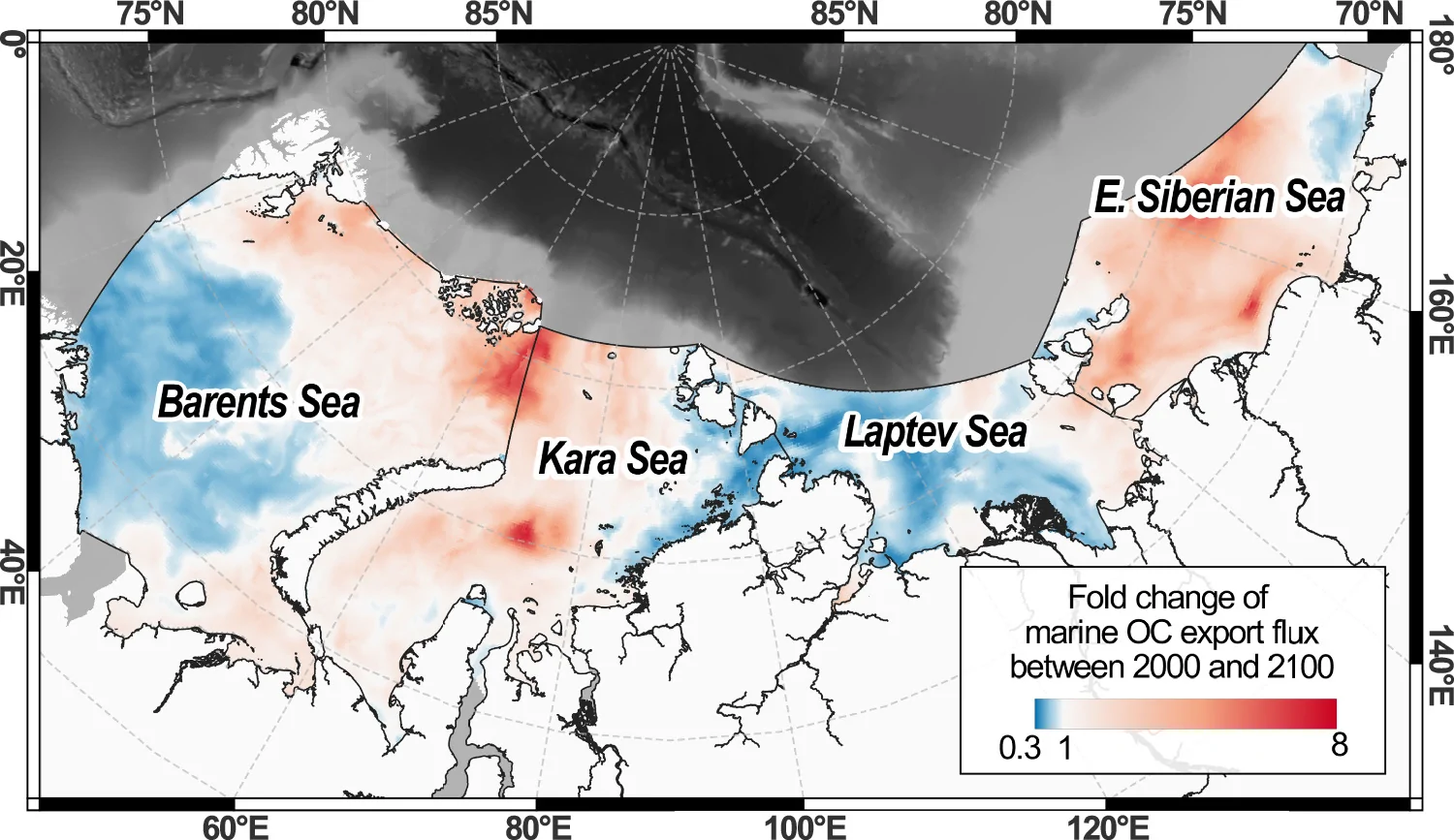
Climate‑driven shifts of marine organic carbon (OC) export fluxes to the sediment bed on Arctic shelves from 2000 to 2100 under the high‑emission Shared Socioeconomic Pathway (SSP) 3-7.0. Increased marine OC export under future climate forcing implies an enhanced capacity of Arctic shelves to remove hydrophobic contaminants from the water column. Results are derived from the global ocean- and sea ice biogeochemistry model FESOM2.1-REcoM3 regridded on a 1/12° resolution mesh^37^. The base map from iBcAOv4^77^, and sea boundaries are from the International Hydrographic Organization^78^. See ref. ^60^ for details of SSP scenarios. The figure was created using QGIS (version 3.40.8).

Under SSP1‑2.6, marine OC export in the Eurasian Arctic shelves remains relatively stable from 2000 to 2100. In contrast, SSP3‑7.0 projects a 54% increase in OC export by 2100, with marine OC export in the East Siberian Sea nearly doubling (Fig. 4). These modeled increases suggest a growing capacity to remove HOCs from the water column in the Eurasian Arctic shelves under the high‑emission climate scenario. Consequently, using the 7 indicator PCBs as model compounds, we estimate that the sediment sink associated with marine OC in 2100 under SSP3-7.0 is about 1.3 times higher than that under SSP1-2.6. This climate-driven increase in the PCB sediment sink can be extrapolated to other HOCs with comparable persistence and physicochemical properties. The Laptev Sea receives large terrestrial OC inputs (Figure S3 in Supplementary Information), predicted to increase by 50% in 2100, and declining marine OC export under SSP3‑7.0 (Fig. 4)^61^, implying reduced HOC removal efficiency by organic matter in the water column, particularly in coastal and estuarine environments. Climate-driven changes in the Arctic may therefore alter the fate of HOCs not only through temperature-dependent physical processes (e.g., re-emission and partitioning) and (a)biotic degradation processes (e.g., bacterial degradation)^8,9^, but also through reshaping the quantity and quality of organic matter that governs contaminant transport in the aqueous phase.

## Methods

### Experimental design

This study was designed to quantitatively and empirically test the enhancement effect of OC origin on HOC concentrations in surface sediments across the full land-to-ocean continuum of OC of the Eurasian Arctic shelves. We measured PCB concentrations in 52 sediment samples and interpreted these data together with published PCB measurements from 59 sites reported in the AMAP assessment (1998) and the MarChem and ICES databases^38–40^. Measured and interpolated values of TOC, δ^13^C, TOC/TN, and sediment mass accumulation rates were obtained from CASCADE^30^. We established empirical relationships between OC‑normalized PCB concentrations and δ^13^C-derived *f*_mar_ in sediment. To investigate variations in the HOC sediment sink in response to climate‑driven shifts in the OC cycle, we coupled extrapolated PCB concentrations in marine OC (ng g^-1^ OC; *f*_mar_ = 100 in Fig. 3) with modeled marine OC export fluxes to sediments for the 21^st^ century derived from the global ocean and sea‑ice biogeochemistry model FESOM2.1‑REcoM3^37^.

### Sampling

Surface sediment samples were collected during multiple expeditions between 2005 and 2020, including The Third Russia-United States cruise in 2005 (Abbreviation: RUS-US; Sediment depth: 2 cm; Sampler: van Veen grab sampler; the same as below)^62^, The International Siberian Shelf Study in 2008 (ISSS-08; 1 cm; GEMAX gravity corer or van Veen grab sampler)^63^, The Swedish-Russian-US Investigation of Carbon-Climate-Cryosphere Interactions in the East Siberian Arctic Ocean in 2014 (SWERUS C-3; 1 cm; Oktopus multicorer)^64^, the Russian–Chinese Expeditions Arctic Silk Way in 2016 (Lavrentiev; 5 cm; multicorer), ARCEx-AMGG Training and Research Cruise in 2018 (1 cm; van Veen grab sampler), the polar front transect expedition in 2018 (2 cm; van Veen grab sampler) and the 82^nd^ expeditionary voyage of the research vessel “Akademik Mstislav Keldysh” in 2020 (1 cm; multicorer)^30^. Detailed sampling information and biogeochemical parameters for each site are provided in Table S1 (Supplementary Dataset) and CASCADE^30^. All samples were frozen throughout the expedition and freeze-dried upon arrival at Stockholm University. Subsamples for PCB analysis were stored in pre-cleaned glass jars.

### PCB analysis

We adopted a validated workflow for wide-scope analysis of HOCs based on gas chromatography-atmospheric pressure chemical ionization-high resolution mass spectrometry (GC-APCI-HRMS)^65^. Briefly, we weighed ~4 g dry sediment into Accelerated Solvent Extraction cells, added 20 µL of a PCB surrogate standard solution in hexane (100 ng mL^−1^ of ^13^C-labeled congeners #32, #47, and #180), and filled up the cells with diatomaceous earth. Extractions were performed using acetone:n‑hexane (1:1, v/v) at 100 °C for three static cycles, yielding ~50 mL of extract per sample. After rotary evaporation, we removed sulfur using activated copper and finally concentrated extracts to ~100 μL under a nitrogen stream.

We injected 3 μL of each sample in pulse splitless mode using a programmed inlet temperature for vaporization to maximize sensitivity, given the low expected concentrations. Compound separation was achieved using a 30 m DB‑5MS Ultra Inert capillary column, with He as carrier gas at a constant flow of 1.5 mL min^–1^. APCI was operated in positive ionization mode under dry conditions, and HRMS in high‑definition MS^E^ mode over a mass range of 100–1200 Da.

We quantified PCB congeners based on the molecular ion of each analyte, using an internal standard method. Concentrations are provided in Table S1 (Supplementary Dataset). We assigned a labeled PCB surrogate to each analyte according to their degree of chlorination. Full details of sample preparation, instrumental settings, and data processing are provided in Section A in Supplementary Information.

We monitored lab contamination via procedural blanks (one per eight samples) using diatomaceous earth as received, which were processed identically to sediment samples. Method detection limits (MDLs) were the sum of the average procedural blank levels (generally negligible) and the instrumental detection limits. We excluded concentrations below MDLs from further analysis. Additionally, we evaluated the data quality and robustness by comparison with published PCB concentrations and congener distributions. See Section B in Supplementary Information for a detailed description of quality assurance and quality control and Table S6 in Supplementary Information for the assigned internal standards, recoveries, and MDLs for each analyte.

### Correction of latitudinal and temporal variations

To isolate the influence of *f*_mar_ on PCB sediment concentrations, we standardized all PCB concentrations to a reference latitude of 75°N and a depositional year of 2008, corresponding to the mean values of the newly measured samples. We corrected the latitudinal decrease of PCB transport based on observed gradients in surface seawater PCB concentrations along a transect from the southern Norwegian Sea to the North Pole (62°–89°N) in 2001. This dataset reported linear changes in seawater concentrations with latitude (*k*_lat.,conc._)^44^. We converted these slopes to relative percentage changes per degree at 75°N (*k*_lat.,percent,75°N_) using the estimated seawater concentration at 75°N (C_water,75°N_) using Eq. 1 (Eq. 1):

1 $${k}_{{{\rm{lat}}}.,{{\rm{percent}}},75^\circ {{\rm{N}}}}=\frac{{k}_{{{\rm{lat}}}.,{{\rm{conc}}}.}}{{{{\rm{C}}}}_{{{\rm{water}}},75^{\circ} {{\rm{N}}}}}\times 100$$

We applied *k*_lat.,percent,75°N_ to correct sediment PCB concentrations (C_i_) to the reference latitude (75°N; Eq. 2):

2 $${{{\rm{C}}}}_{{{\rm{i}}},75}=\frac{{{{\rm{C}}}}_{{{\rm{i}}}}}{\left(1+{k}_{{{\rm{lat}}}.,{{\rm{precent}}},75^\circ {{\rm{N}}}}\times \left({{\rm{Latitude}}}-75\right)/100\right)}$$

We used latitudinal gradients in surface water PCB concentrations as a proxy for temperature to correct the influence of temperature on PCB transport. The gradients were observed in 2001, while the sample collection year in this study ranges from 1994 to 2018. Temperature variations from 1994 to 2018 in the Arctic could bring uncertainty to the current correction.

We corrected temporal variation based on depositional years (year_dep._) using long‑term trends in atmospheric PCB concentrations measured at the Zeppelin station (Ny‑Ålesund, Svalbard) from 1993 to 2012^41^. We converted reported half-life (t_1/2_; time required for a 50% decrease in air concentration) to annual change rate (*k*_year_; Eq. 3):

3 $${k}_{{{\rm{year}}}}=\frac{{\mathrm{ln}}(1/2)}{{{{\rm{t}}}}_{1/2}}$$

We applied *k*_year_ to correct sediment concentrations to the reference depositional year (year_dep._) of 2008 as Eq. 4:

4 $${{{\rm{C}}}}_{{{\rm{i}}},2008}={{{\rm{C}}}}_{{{\rm{i}}}}\times {e}^{{k}_{{{\rm{year}}}}\left({{{\rm{year}}}}_{{{\rm{dep.}}}}-2008\right)}$$

The depositional year was estimated from sampling year, sediment depth, and mass accumulation rates (MAR). MAR values were interpolated from the CASCADE database using ordinary kriging^66^. We assume the porosity and dry density for all samples are 0.7 and 2.65 g cm^−3^, respectively, consistent with reported data for sediment cores in East Siberian Arctic Shelves^63^. We acknowledge that we neglected the potential uncertainty from turbation during sampling. Both trend parameters are listed in Table S6 in Supplementary Information. For congeners lacking compound‑specific parameters, we assume parameters for isomers are the same.

### Compilation of the PCB database

We combined site‑specific PCB concentrations for the Eurasian Arctic shelves from the AMAP assessment report (1998) and MarChem and ICES databases^38–40^. The MarChem and ICES datasets provide congener‑specific PCB concentrations, predominantly from the Barents Sea. For the MarChem data, we excluded sites exhibiting anomalously high contributions of PCB28 and PCB180 that were inconsistent with both our newly measured data and ICES records, and that did not follow the westward decrease in the relative contribution of PCB28 across the Eurasian Arctic shelves. For ICES data, sites reporting concentrations that were one order of magnitude lower than those from comparable locations and sampling years were excluded. Detailed data screening criteria and quality control procedures are described in Section B in Supplementary Information.

The AMAP assessment report (1998) provides only sum concentrations for PCBs. To enable congener‑specific analyses, we disaggregated these sum values into congener-specific values using an iterative, data‑constrained approach^39^. First, we established empirical relationships between *f*_mar_ and available congener‑specific PCB concentrations in sediment, using both newly generated and literature data. Subsequently, we estimated theoretical congener profiles at a given latitude, depositional year, and *f*_mar_ in sediment, allowing partition of Σ₇PCB concentrations into congener-specific values. The reconstructed congener‑specific concentrations were subsequently included in regression analyses with *f*_mar_ in sediment, generating updated regression slopes. These updated relationships were then used to recalculate congener profiles and to repeat the fraction procedure. We continued the iteration until successive regression slopes converged, i.e., relative changes of slope less than 1% between iterations for all congeners. We only included the final estimated congener‑specific concentrations in establishing functions between *f*_mar_ and PCB concentrations in sediment (Fig. 3), to maximize coverage of geographical space and land-to-ocean continuum of OC. Notably, excluding these estimated concentrations does not alter the direction of the observed associations (Fig. 2).

### OC source apportionment

We estimated the fraction of terrestrial and marine OC (*f*_ter_ and *f*_mar_) using an end-member mixing model based on δ^13^C (Eq. 5), assuming these as the dominant OC sources (*f*_ter_ + *f*_mar_ = 1)^26^:

5 $${{\rm{\delta }}}^{13}{{\rm{C}}}={{\rm{\delta }}}^{13}{{\rm{C}}}_{{\rm{ter}}} \times {{f}}_{{\rm{ter}}}+{{\rm{\delta}}}^{13}{{{\rm{C}}}}_{{\rm{mar}}}\times {f}_{{\rm{mar}}}$$

where δ^13^C_ter_ and δ^13^C_mar_ represent the end‑members for terrestrial and marine OC, respectively.

We selected end-member values according to an extensive literature synthesis of Arctic OC sources^29^. Open-marine δ^13^C_mar_ values (-21‰), representative of phytoplankton and algae influenced by Atlantic or Pacific water‑mass inflow, were applied to the Barents Sea and the East Siberian Sea east of 160°E. For the Laptev Sea, the Kara Sea, and the East Siberian Sea west of 160°E, we applied a more depleted Arctic marine phytoplankton value (-23.2‰), reflecting regionally compiled measurements from the central Arctic Ocean.

Terrestrial OC in the study region originates from multiple sources in addition to surface soils (-26.6 ± 1.7‰), including petrogenic carbon in the Barents Sea (-26.0 ± 2.0‰), peat‑derived OC in the Kara Sea (-26.6 ± 1.7‰), and ice‑complex deposits in the Laptev and East Siberian Seas (-26.3 ± 0.7‰). Accordingly, region‑specific terrestrial end‑members were applied: -26.3‰ for the Barents Sea, -26.6‰ for the Kara Sea, and -26.5‰ for the Laptev and East Siberian Seas.

### Estimation of marine OC export fluxes to sediment and the PCB sink

We estimated marine OC export fluxes to sediment for the 21^st^ century using simulations from the global ocean–sea‑ice biogeochemistry model FESOM2.1‑REcoM3 regridded on a regular 1/12° resolution mesh^37^. FESOM is a global ocean circulation model using an unstructured mesh with an original resolution of 4.5 km at pan-Arctic scale, which includes a dynamic-thermodynamic sea ice model^67–69^. This ‘state-of-the-art’ version has been specifically assessed and tuned for the Arctic Ocean in single-model studies^70^, in Model Intercomparison Projects^71,72^, and included in the AWI (Alfred Wegener Institute, Germany) Climate Model^73,74^. The Regulated Ecosystem Model v.3 (REcoM3), a biogeochemical model of intermediate complexity^75^, resolves the biogeochemical fluxes of carbon, nitrogen, silicon, iron, and oxygen with a total of 28 prognostic tracers, including the full carbonate chemistry and air–sea CO_2_ exchange using the mocsy 2.0 scheme^76^. The simulations encompass one historical run (2000-2014) and projection experiments of two climate change scenarios (i.e., SSP1-2.6 and 3-7.0)^60^. The simulations also represent the varying inputs of carbon and nutrients from rivers and coastal erosion. See ref. ^33^ for the details of model setup and evaluation.

We estimated PCB concentrations in marine OC from the empirical relationships between standardized OC‑normalized PCB concentrations and δ¹³C‑derived *f*_mar_ in sediment (Fig. 3). Specifically, we define PCB concentrations per unit marine OC (C_marine_, _75°N_,_2008_; ng g⁻¹OC) as the extrapolated value at *f*_mar_ = 100%, standardized to 75°N and the depositional year 2008. The quantity of PCBs associated with marine OC in sediments (PCB sequestration amount_i,j,k_) was calculated by multiplying C_marine_, _75°N_,_2008_ by the modeled marine OC export flux (OC flux_i,j,k_) for each grid cell and depositional year (Eq. 6):

6 $${{\rm{PCB}}}\; {{\rm{sequestration}}}\; {{\rm{amount}}}_{{{\rm{i}}},{{\rm{j}}},{{\rm{k}}}}={{{\rm{C}}}}_{{{\rm{marine}}},75,2008}\times {{{\rm{OC\; flux}}}}_{{{\rm{i}}},{{\rm{j}}},{{\rm{k}}}}$$

where i, j and k denote the grid cell, latitude, and depositional year, respectively. We back-corrected the resulting PCB sink estimates for site‑specific latitude and depositional year using the correction procedures described above.

The uncertainty of modeled export fluxes of marine OC has previously been evaluated in ref. ^37^. Briefly, scenario uncertainty can be quantified by the difference between SSP3-7.0 and SSP1-2.6. Inter-model uncertainty was evaluated through comparison with the Coupled Model Intercomparison Project Phase 6 (CMIP6) ensemble. Comparison of OC export fluxes from the current model with those from 16 to 17 CMIP6 Earth System Models demonstrated consistent dynamics. The modeled OC export flux was also compared with the most comprehensive current observational dataset for the sinking of particulate organic matter. The modeled OC export flux was able to reproduce observations significantly and reasonably well, with a noticeable improvement for the Arctic Ocean^37^.

To demonstrate changes in marine OC export fluxes and in the associated capacity to remove HOCs from the water column in Eurasian Arctic shelves, we applied the fixed PCB concentrations per marine OC and fixed temporal and latitudinal corrections for annual export fluxes of PCBs to sediment throughout the 21^st^ century under both low- and high-emission scenarios. The uncertainty of the PCB sink ratio associated with marine OC between the two scenarios mainly lies in the modeled marine OC export fluxes. Since temperature is a strong factor influencing both volatilization and global fractionation of HOCs^9^, the fixed temporal and latitudinal corrections cannot fully reflect the influence of temperature change in the two modeled scenarios. Therefore, the current estimations cannot be interpreted as predictions of the magnitude of the PCB sink in Arctic shelf sediments.

### Statistical analysis

We replaced the non-detected values with half MDLs when estimating means for individual compounds. When MDLs were not available, the non-detected values were replaced by half of the minimum values. Non-detected values were not included in the regression analysis. We tested differences in standardized OC-normalized concentrations between two groups using the Wilcoxon signed-rank test, considering limited sample sizes. We tested associations between concentrations and sampling year using Spearman correlation analysis, and other associations with Pearson correlation analysis. We established basic linear regressions between *f*_mar_ and standardized OC-normalized concentrations in sediment after log-transformation. To compare OC export fluxes and the PCB sink in 2000 and 2100, and those in 2100 under different scenarios, we established temporal trends using linear regression with lag-1 autocorrelation to extrapolate values in 2000 and 2100. For all analyses, *p*-values less than 0.05 were considered as significant.

## Supplementary information

Supplementary information

Description of Additional Supplementary File

Transparent Peer Review file

Supplemntary Dataset

## Data Availability

All data generated or analyzed during this study are included in this published article and its Supplementary Information files. The data underlying figures in the main text are available on the Zenodo repository under the DOI identifier: https://doi.org/10.5281/zenodo.22168380.
