# Supplementary material for: Marine primary production enhances removal of hydrophobic organic contaminants from Eurasian Arctic shelf waters
Source: Nat Commun. 2026 Oct 23;17:11074. doi: 10.1038/s41467-026-78857-3 (PMC13694705; doi:10.1038/s41467-026-78857-3)
Supplement: Supplementary file 1 [file 41467_2026_78857_MOESM1_ESM.pdf]

# Supplementary Information

# Marine Primary Production Enhances Removal of Hydrophobic Organic Contaminants from Eurasian Arctic Shelf Waters


Xiaodi Shi (史咲頔)[1*], Laurent Oziel[2,3], Jonathan P. Benskin[1], Örjan Gustafsson[1,4], Anna Sobek[1*]

1. Department of Environmental Science, Stockholm University, Stockholm, Sweden
2. Alfred-Wegener-Institut - Helmholtz-Zentrum für Polar- und Meeresforschung, Bremerhaven, Germany
3. CNRM, Université de Toulouse, Météo-France, CNRS, Toulouse, France
4. The Bolin Centre for Climate Research, Stockholm University, Stockholm, Sweden

*Corresponding authors: xiaodi.shi@aces.su.se, anna.sobek@aces.su.se

**Contents:**


**Section A.** Polychlorinated biphenyl analysis.

**Section B.** Quality assurance and quality control.

**Figure S1.** Congener distribution of seven indicator polychlorinated biphenyls in sediment and in polar mixed layer.

**Figure S2.** Organic carbon-normalized concentrations of seven polychlorinated biphenyl congeners in the surface sediment collected in Barents and Kara Seas after correction of latitudinal and temporal variations.

**Figure S3.** Coastal erosion in 2100 under Shared Socioeconomic Pathway 3-7.0.

**Table S1.** Sampling and biogeochemistry information and concentrations of polychlorinated biphenyls in sediment samples measured in the current study. (Supplementary Dataset)

**Table S2.** Sampling and biogeochemistry information and concentrations of polychlorinated biphenyls in sediment samples in literature. (Supplementary Dataset)

**Table S3.** Summary of polychlorinated biphenyl concentrations from relevant literature.

**Table S4.** Summary of regression between polychlorinated biphenyl concentrations and fraction of marine organic carbon in sediment.

**Table S5.** Time series of export fluxes of marine organic carbon to sediment beds and the ratio of polychlorinated biphenyl sink associated with marine organic carbon between two scenarios. (Supplementary Dataset)

**Table S6.** Quantitative information and trend parameters of polychlorinated biphenyls.

**Supplementary References.**

**Section A. Polychlorinated biphenyl (PCB) analysis.** We adopted a previously validated workflow for wide-scope targeted analysis of hydrophobic organic contaminants.[1] Extraction was performed using an accelerated solvent extraction system (ASE 350; Dionex, U.S.A.). We pre-cleaned the 34 mL extraction cells using 20 mL of acetone/hexane (ACE/HEX; 1:1, v/v) at 100 °C for 10 min. Empty cells were dried in a clean fume hood at room temperature overnight. We loaded the cells sequentially with a pre-baked glass fiber filter (GFF; Dionex, Thermo Scientific), diatomaceous earth (DE; Dionex, Thermo Scientific), approximately 4 g of freeze-dried sediment, DE, and a GFF. Samples were fortified with 20 μL of a PCB surrogate standard solution in hexane (100 ng $mL^{-1}$ of $^{13}C$-labeled congeners #32, #47, and #180) and were extracted three times, each time with approximately 17 mL of ACE/HEX (1:1, v/v) at 100 °C for 10 min. The system was washed three times with 5 mL of solvent between samples. Extracts were concentrated to approximately 1 mL using a rotary evaporator at 30 °C, transferred to clean test tubes, and amended with 1 mL of activated copper in hexane to remove sulfur. After vortexing and centrifugation, the supernatant was collected into a clean amber glass bottle, reduced to approximately 100 μL under a gentle nitrogen stream, and transferred to sample vials. All glassware and GFFs were baked at 450 °C for 4 h. All metal parts and tools were ultrasonicated using ACE for 20 min. Potential photolysis was prevented by wrapping containers with aluminum foil or using amber glassware.

We employed a Waters quadrupole-cyclic ion mobility-time-of-flight mass spectrometer (Waters Corp., Wilmslow, U.K.) coupled to an Agilent 8890 gas chromatograph (GC; Agilent Technologies, Santa Clara, CA, U.S.A) via an atmospheric pressure chemical ionization (APCI) source for analysis. 3 μL of sample was injected in pulse splitless mode using programmed temperature vaporization (i.e., initially 100 °C for 0.15 min, increased at 600 °C $min^{-1}$ to 280 °C, and held for 1 min). Analytes were separated on a 30-m DB-5MS Ultra Inert column (i.d., 0.25 mm; film thickness, 0.25 μm; Agilent Technologies) using helium as the carrier gas at a constant flow rate of 1.5 mL $min^{-1}$. The GC oven temperature program was as follows: hold at 70 °C for 1 min; increase at 10 °C $min^{-1}$ to 310 °C, and then hold for 15 min.

The APCI source was operated in positive ion mode under dry conditions. The transfer line and ion source temperatures were maintained at 290 °C and 150 °C, respectively. The corona

discharge current and cone voltage were set to 2 μA and 30 V, respectively. Nitrogen was used as the makeup, auxiliary, and cone gas at flow rates of 200 mL $min^{-1}$, 350 L $h^{-1}$, and 250 L $h^{-1}$, respectively, under dry conditions. The mass spectrometer was operated in high-definition $MS^E$ mode over a mass range of 100–1200 amu. Collision energy was fixed at 6 eV in low-energy mode and ramped from 15 to 50 eV in high-energy mode. The scan time was 0.3 s for each mode.

Analytes were quantified based on their molecular ions in the full-scan mass spectrum using an internal standard method, and peaks were integrated with a mass tolerance window of 50 ppm using MassLynx software (version 4.2, Waters Corp.). Labeled PCB standards were assigned to analytes based on their degree of chlorination.

**Section B. Quality assurance and quality control.** For new polychlorinated biphenyl (PCB) measurements as part of the present work, we burned all glassware and quartz filters at 450 °C for 4 h and ultrasonicated all metal parts for 20 min in acetone. Except for PEEK seals used for accelerated solvent extraction and caps for sample vials, we did not use plastic items during sample preparation. To prevent potential photolysis, we wrapped containers in aluminum foil or used amber glassware. We monitored lab contamination using procedural blanks (one per eight samples) prepared with diatomaceous earth as received and subjected to the same preparation procedures as the sediment samples. Method detection limits (MDLs) are the sum of the average amounts measured in blank samples (which were negligible) and instrumental detection limits (see Table S6 in Supplementary Information for MDLs). We removed concentrations lower than MDLs from further analysis.

The accuracy and precision of the method were evaluated in our previous study.[1] Briefly, a comparison of triplicate measurements of NIST standard reference material (SRM 1941b-Organics in Marine Sediment) to certified concentrations revealed an average accuracy for PCBs of 100% with a standard deviation (SD) of 18.8%. Further, spike/recovery experiments using a Baltic Sea sediment sample (58.26° N, 16.91° E) produced average recoveries for the seven indicator PCBs of 103% (SD: 13.4%; Table S6 in Supplementary Information).

All newly generated sum concentrations of seven indicator PCBs ($\Sigma_7$PCB concentrations) were less than 1 ng $g^{-1}$ dry weight (d.w.). A previous summary of PCB concentrations in Arctic circumpolar marine surface sediments (AMAP Arctic Monitoring and Assessment Programme assessment report (1998)) showed that most investigated sites had sum concentrations below 1 ng $g^{-1}$ d.w., despite differences in sampling years, numbers of congeners, and analytical techniques.[2] The average $\Sigma_7$PCB concentrations were 151 pg $g^{-1}$ d.w. (SD: 26.6 pg $g^{-1}$ d.w.) in the Barents Sea, 157 pg $g^{-1}$ d.w. (SD: 88.0 pg $g^{-1}$ d.w.) in the Kara Sea, 167 pg $g^{-1}$ d.w. (SD: 105 pg $g^{-1}$ d.w.) in the Laptev Sea, and 144 pg $g^{-1}$ d.w. (SD: 108 pg $g^{-1}$ d.w.) in the East Siberian Sea. These concentrations are of the same order of magnitude as previously reported values for the Barents Sea (10-4100 pg $g^{-1}$ d.w.) and Kara Sea (10-7800 pg $g^{-1}$ d.w.)[2-4] as well as for other parts of the Arctic Ocean (e.g., 10-1005 pg $g^{-1}$ d.w. in the Chukchi Sea and 80-800 pg $g^{-1}$ d.w. in the Beaufort Sea; See Table S3 in the Supplementary Dataset for a summary of previous

PCB measurements in Arctic surface sediments).[2,5,6]

Additionally, we assessed the comparability and variability of the combined dataset from multiple sources in terms of temporal and spatial patterns. $\Sigma_7$PCB concentrations in each shelf sea within the combined database are of the same order of magnitude. Some sites from ICES (International Council for the Exploration of the Sea) were excluded because their $\Sigma_7$PCB concentrations were one order of magnitude lower than those at sites in similar locations and sampling years.[7] $\Sigma_7$PCB concentrations in each shelf sea show a significant decreasing trend with sampling year (1994–2020), consistent with reduced transport of PCBs to the Arctic following the cessation of their production.[8]

PCB 28 was the dominant congener, with its contribution decreasing from east (61% in the East Siberian Sea) to west (19% in the Barents Sea), indicating a major influence of atmospheric transport of more volatile PCB congeners.[2] Hexa- and hepta-PCBs exhibited an opposite trend, collectively contributing 15% in the East Siberian Sea and 36% in the Barents Sea, reflecting the closer proximity of western shelf seas to former high-consumption regions in northeastern North America and northern Europe.[9] Congener profiles in surface sediments from the combined database are consistent with previously reported profiles for the polar mixed layer in each shelf sea (See Figure S1 in Supplementary Information).[10] Some sites in MarChem (The Marine Chemistry database for Norwegian waters) showed higher contributions of PCB 28 and PCB 180. PCBs in the MarChem dataset were measured using gas chromatography with an electron capture detector.[11] Interfering or co-eluting electrophilic compounds can therefore compromise accurate quantification of target analytes.[12] The current study employed high-resolution mass spectrometry to enhance analytical specificity. Consequently, MarChem sites with elevated contributions of PCB 28 and 180 were excluded, as their congener patterns differed from both our measurements and ICES data and did not follow the observed east-to-west decrease in PCB 28 contribution. Overall, the agreement between observed temporal and longitudinal patterns and prior knowledge supports the reliability of the present database.

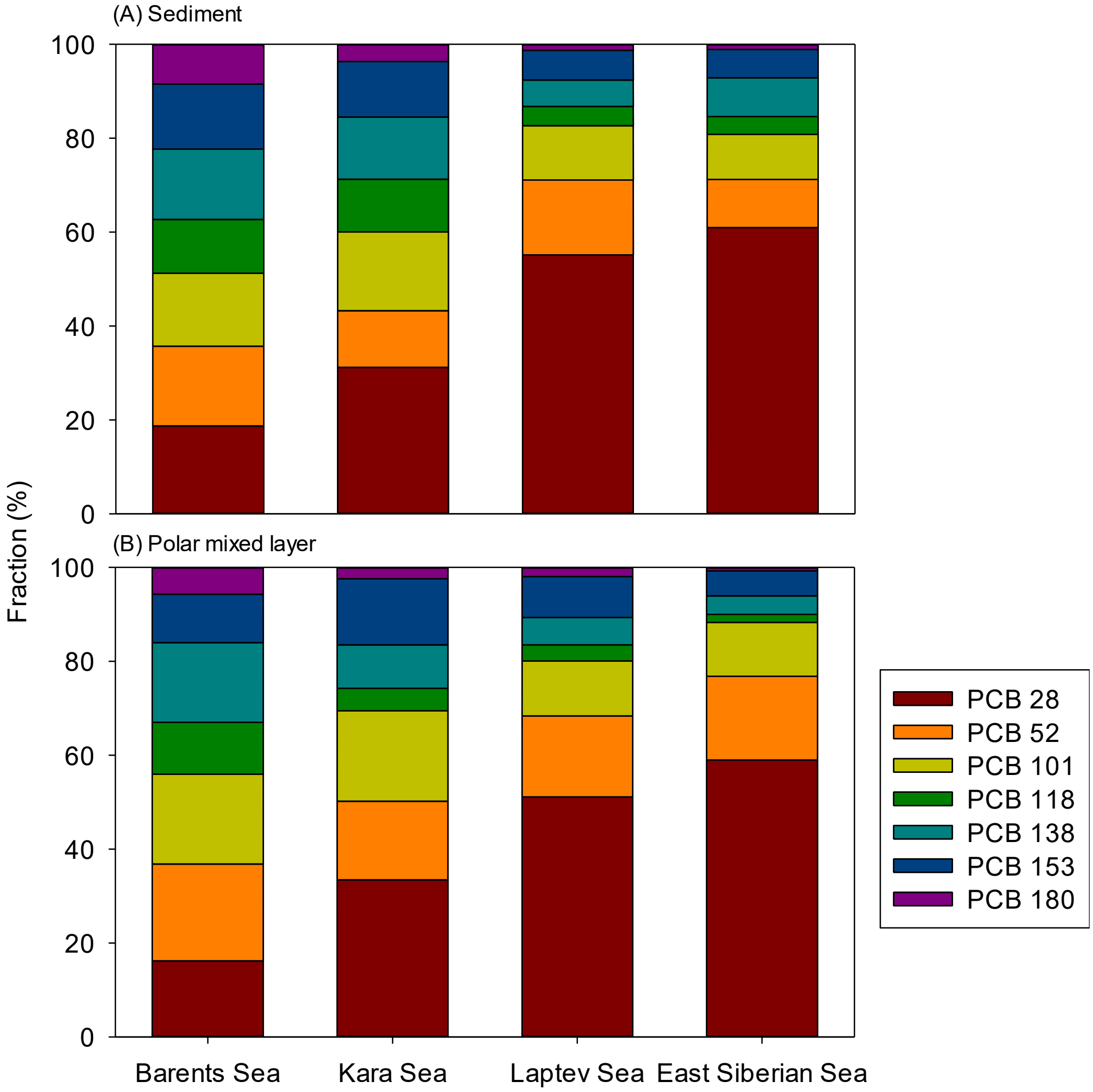


**Figure S1. Congener distributions of seven indicator polychlorinated biphenyls (PCB) in (A) sediment and (B) polar mixed layer.** Congener distributions in sediment were calculated based on the current combined database, and for polar mixed layer ref. 10.

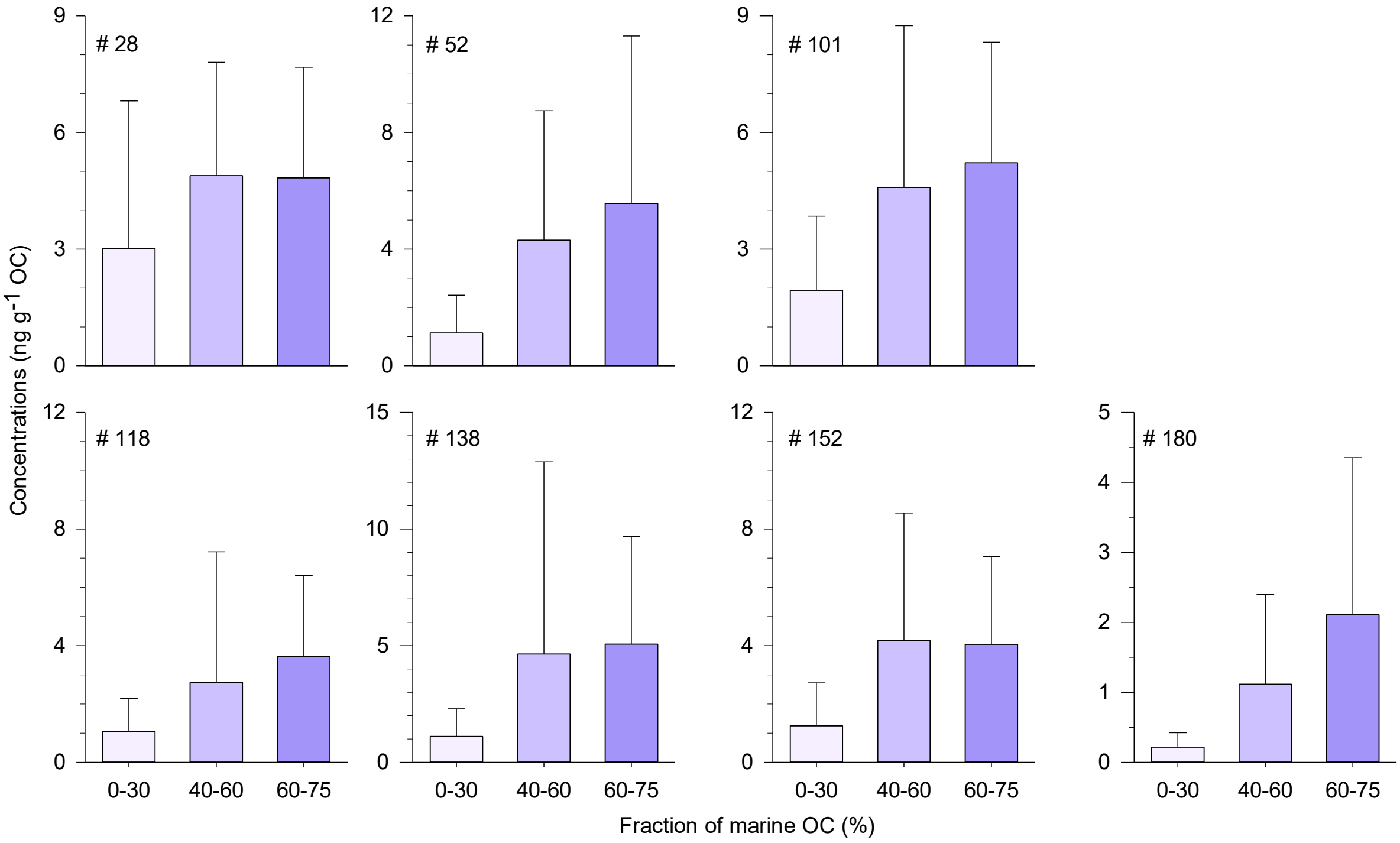


**Figure S2. Organic carbon (OC)-normalized concentrations (ng g$^{-1}$ OC) of seven polychlorinated biphenyl congeners in the surface sediment collected in Barents and Kara Seas after correction of latitudinal and temporal variations.** After correction, all samples were standardized to a latitude of 75º N and a deposition year of 2008. The bars and whiskers represent average concentrations and standard deviations (n = 47), respectively. Colors from bright to dark indicate fraction of marine OC from low to high.

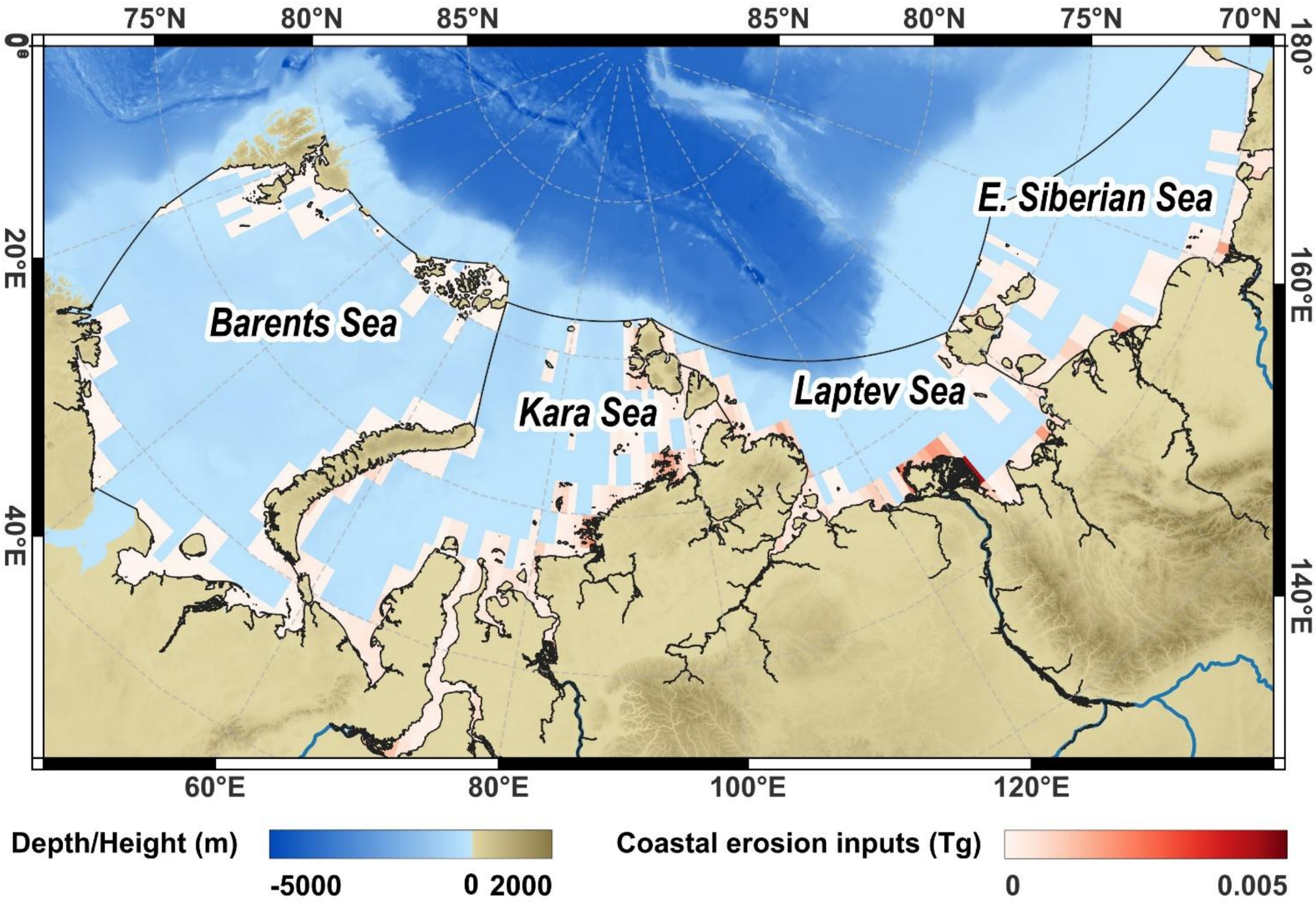


**Figure S3. Coastal erosion in 2100 under Shared Socioeconomic Pathway 3-7.0.** The estimation is adjusted from a long-term observational dataset (ref. 13). The figure was created using QGIS (version 3.40.8).

**Table S3. Summary of polychlorinated biphenyl (PCB) concentrations from relevant literature.** NA: Not available.

| Entry | Region | Sampling year | Number of congeners | Average | Standard deviation | Median | Range | Reference |
|---|---|---|---|---|---|---|---|---|
| **Seven indicator PCB concentrations (pg $g^{-1}$ dry weight; including PCB 28, 52, 101, 118, 138, 153 and 180)** | | | | | | | | |
| 1 | Greenland Sea | 2018 | 7 | 249 | 37.6 | 243 | 180-321 | |
| 2 | Greenland Sea | 2019 | 7 | 253 | 46.9 | 242 | 207-347 | |
| 3 | Greenland Sea | 2020 | 7 | 273 | 55.1 | 276 | 190-360 | 14 |
| 4 | Greenland Sea | 2021 | 7 | 280 | 28.3 | 280 | 233-316 | |
| 5 | Greenland Sea | 2022 | 7 | 281 | 36.4 | 272 | 245-360 | |
| 6 | Greenland Sea | 2018 | 7 | 267 | 349 | 82 | 11.1-1254 | 15 |
| 7 | Greenland Sea | 2015 | 7 | 1036 | 754 | 866 | 100-2381 | |
| 8 | Greenland Sea | 2016 | 7 | 517 | 284 | 443 | 170-1005 | 16 |
| 9 | Greenland Sea | 2017 | 7 | NA | NA | 228 | 148-307 | |
| 10 | Greenland Sea | 2017-2022 | 7 | 521 | 460 | 310 | 90-1450 | |
| 11 | Norwegian Sea | 2006-2021 | 7 | 265 | 213 | 220 | 20-910 | 17 |
| 12 | Arctic Ocean | 2018-2022 | 7 | 377 | 290 | 345 | 30-1160 | |
| 13 | Greenland Sea | 1992-2009 | 7 | 4323 | 6683 | 1233 | 9.47-26900 | 7 |
| 14 | Norwegian Sea | 1992-2009 | 7 | 39400 | 70500 | 13100 | 138-438000 | |
| **Sum concentrations (pg $g^{-1}$ dry weight)** | | | | | | | | |
| 15 | Barents Sea | 1997 | 11 | 2100 | 1700 | NA | 1060-4100 | 3 |
| 16 | Kara Sea | 1993 | 55 | 410 | 340 | NA | NA | 4 |
| 17 | Kara Sea | 1994 | NA | 856 | 547 | 810 | 210-7800 | 7 |
| 18 | Barents Sea | <1998 | NA | 470 | 422 | 250 | 10-1670 | |
| 19 | Kara Sea | <1998 | NA | 426 | 467 | 250 | 10-2200 | |
| 20 | Arctic Ocean | <1998 | NA | 289 | 166 | 250 | 80-690 | |
| 21 | Greenland Sea | <1998 | NA | 5130 | 9600 | 595 | 210-31430 | 2 |
| 22 | Norwegian Sea | <1998 | NA | 1370 | 1150 | 1060 | 260-3650 | |
| 23 | White Sea | <1998 | NA | 1240 | 824 | 1250 | 250-2500 | |
| 24 | Beaufort Sea | <1998 | NA | 302 | 259 | 230 | 80-800 | |
| 25 | Greenland Sea | 2018 | 34 | 330 | 60 | NA | NA | |
| 26 | Greenland Sea | 2019 | 34 | 330 | 60 | NA | NA | |
| 27 | Greenland Sea | 2020 | 34 | 390 | 110 | NA | NA | 14 |
| 28 | Greenland Sea | 2021 | 34 | 390 | 70 | NA | NA | |
| 29 | Greenland Sea | 2022 | 34 | 400 | 60 | NA | NA | |
| 30 | Greenland Sea | 2018 | 14 | NA | NA | 98 | 12-1600 | 15 |
| 31 | Chukchi Sea | 2008 | 14 | 190 | NA | NA | 60-640 | 5 |
| 32 | Canadian Basin | 2008 | 14 | 150 | NA | NA | 24-600 | |
| 33 | Chukchi Sea | 2010 | 46 | 387 | NA | NA | 124-1005 | |
| 34 | Chukchi Sea | 2012 | 46 | 185 | NA | NA | 10-357 | 6 |
| 35 | Canadian Basin | 2012 | 46 | 149 | NA | NA | 24-330 | |
| 36 | Saglek Bay | 1999 | 55 | 650 | NA | NA | 270-1650 | 18 |
| 37 | Hudson Bay | 2005 | NA | NA | NA | NA | 40-150 | 19 |

**Table S4. Summary of regressions between polychlorinated biphenyl concentrations and fractions of marine organic carbon in sediment.**

| Substances | Estimated PCB loading (ng $g^{-1}$ OC) | | $p$ | $R^2$ |
|---|---|---|---|---|
| | $f_{mar}$ = 0 | $f_{mar}$ = 100 | | |
| **Barents and Kara Seas** | | | | |
| PCB 28 | 0.98 | 5.87 | <0.01 | 0.26 |
| PCB 52 | 0.40 | 4.41 | <0.01 | 0.20 |
| PCB 101 | 0.78 | 6.04 | <0.01 | 0.30 |
| PCB 118 | 0.39 | 3.86 | <0.01 | 0.30 |
| PCB 138 | 0.43 | 4.72 | <0.01 | 0.27 |
| PCB 153 | 0.49 | 4.95 | <0.01 | 0.31 |
| PCB180 | 0.096 | 0.82 | <0.01 | 0.28 |
| **Laptev and East Siberian Seas** | | | | |
| PCB 28 | 4.04 | 9.39 | 0.024 | 0.080 |
| PCB 52 | 0.74 | 1.14 | 0.49 | 0.0092 |
| PCB 101 | 0.43 | 3.41 | <0.01 | 0.33 |
| PCB 118 | 0.17 | 1.53 | <0.01 | 0.34 |
| PCB 138 | 0.10 | 2.64 | <0.01 | 0.44 |
| PCB 153 | 0.13 | 3.18 | <0.01 | 0.49 |
| PCB 180 | 0.05 | 0.44 | <0.01 | 0.26 |

**Abbreviations:** OC: Organic carbon; PCB: Polychlorinated biphenyls; $f_{mar}$: Fraction of marine OC in sediment

**Table S6. Quantitative information and trend parameters of polychlorinated biphenyls (PCBs).**

| Substances | Internal standards | Method detection limits ($pg\ g^{-1}$ dry weight)[a] | Recoveries (%)[b] | | Trends parameter | |
|---|---|---|---|---|---|---|
| | | | Mean | Standard deviation | Temporal ($t_{1/2}$; year)[8] | Latitude (% $degree^{-1}$)[20] |
| PCB 28 | $^{13}C_{12}$-PCB 32 | 1.83 | 77.4 | 13.7 | 8.2 | 0 |
| PCB 52 | $^{13}C_{12}$-PCB 47 | 3.15 | 116 | 20.9 | 4.4 | -3.7 |
| PCB 101 | $^{13}C_{12}$-PCB 47 | 1.40 | 100 | 9.81 | 11 | -11 |
| PCB 118 | $^{13}C_{12}$-PCB 47 | 2.83 | 110 | 14.1 | 6.1 | -11 |
| PCB 138 | $^{13}C_{12}$-PCB 47 | 0.96 | 106 | 13.8 | 5.1 | -12 |
| PCB 153 | $^{13}C_{12}$-PCB 47 | 1.58 | 116 | 21.4 | 6.2 | -12 |
| PCB 180 | $^{13}C_{12}$-PCB 180 | 1.58 | 98.7 | 10.2 | 4.4 | -0.37 |

**Notes:**

[a.] Method detection limits are average amount in procedure blank samples plus instrumental detection limits. Instrumental detection limits were calculated by t × relative standard deviation (%) × the lowest detectable concentration of five replicates.

[b.] Recoveries are reported in ref. 1.

**Supplementary References:**


1. Shi, X., Langberg, H. A., Sobek, A. & Benskin, J. P. Exploiting molecular ions for screening hydrophobic contaminants in sediments using gas chromatography-atmospheric pressure chemical ionization-ion mobility-mass spectrometry. *Environ. Sci. Technol.* **59**, 4699–4708 (2025).

2. Arctic Monitoring and Assessment Programme. *AMAP Assessment Report: Arctic Pollution Issues.* (1998).

3. Savinov, V. M., Savinova, T. N., Matishov, G. G., Dahle, S. & Næs, K. Polycyclic aromatic hydrocarbons (PAHs) and organochlorines (OCs) in bottom sediments of the Guba Pechenga, Barents Sea, Russia. *Sci. Total Environ.* **306**, 39–56 (2003).

4. Sericano, J. L. Brooks, J. M., Champ, M. A., Kennicutt II, M. C. & Makeyev, V. V. Trace contaminant concentrations in the Kara Sea and its adjacent rivers, Russia. *Mar. Pollut. Bull*. **42**, 1017–1030 (2001).

5. Hong, Q. et al. Occurrence of polychlorinated biphenyls (PCBs) together with sediment properties in the surface sediments of the Bering Sea, Chukchi Sea and Canada Basin. *Chemosphere*. **88**, 1340–1345 (2012).

6. Ma, Y. et al. Persistent organic pollutants in ocean sediments from the North Pacific to the Arctic Ocean. *J. Geophys. Res. Oceans.* **120**, 2723–2735 (2015).

7. ICES. International Council for the Exploration of the Sea (ICES) dataset. https://www.ices.dk/data/dataset-collections/Pages/default.aspx.

8. Arctic Monitoring and Assessment Programme. *AMAP Assessment 2015: Temporal Trends in Persistent Organic Pollutants in the Arctic.* (2015).

9. Breivik, K., Sweetman, A., Pacyna, J. M. & Jones, K. C. Towards a global historical emission inventory for selected PCB congeners - A mass balance approach-3. An update. *Sci. Total Environ.* **377**, 296307 (2007).

10. Carrizo, D. & Gustafsson, Ö. Distribution and inventories of polychlorinated biphenyls in the polar mixed layer of seven Pan-Arctic shelf seas and the interior basins. *Environ. Sci. Technol.* **45**, 1420–1427 (2011).

11. Jensen, H. Banica, A. & Boitsov, S. *Mareano Kjemiprogram: Metodebeskrivelse for prøvetaking og analyse av havbunnssedimenter*. (2024).

12. Bianco, G. et al. Polychlorinated biphenyls in contaminated soil samples evaluated by GC–ECD with dual-column and GC–HRMS. *Chemosphere* **73**, 104–112 (2008).

13. Terhaar, J., Lauerwald, R., Regnier, P., Gruber, N. & Bopp, L. Around one third of current Arctic Ocean primary production sustained by rivers and coastal erosion. *Nat. Commun.* **12**, 169 (2021).

14. Pescatore, T., Rauseo, J., Spataro, F., Calace, N. & Patrolecco, L. Persistent organic pollutants (POPs) in marine sediments of the Arctic fjord Kongsfjorden, Svalbard Islands. *Mar. Pollut. Bull.* **211**, 117407 (2025).

15. Johansen, S., Poste, A., Allan, I., Evenset, A. & Carlsson, P. Terrestrial inputs govern spatial distribution of polychlorinated biphenyls (PCBs) and hexachlorobenzene (HCB) in an Arctic fjord system (Isfjorden, Svalbard). *Environ. Pollut.* **281**, 116963 (2021).

16. Pouch, A., Zaborska, A., Legeżyńska, J., Deja, K. & Pazdro, K. Assessment of exposure of benthic organisms to selected organochlorine pollutants in the west Spitsbergen fjords. *Sci. Total Environ.* **896**, 165262 (2023).

17. The Geological Survey of Norway & the Institute of Marine Research. The Marine Chemistry database for Norwegian waters. https://marchem-editor.hi.no.

18. Kuzyk, Z.A., Stow J.P., Burgess, N.M., Solomon, S.M. & Reimer, K.J. PCBs in sediments and the coastal food web near a local contaminant source in Saglek Bay, Labrador. *Sci. Total Environ.* **351-352**, 264–284 (2005).

19. Kuzyk, Z. Z. A., Macdonald, R. W., Johannessen, S. C. & Stern, G. A. Biogeochemical controls on PCB deposition in Hudson Bay. *Environ. Sci. Technol.* **44**, 3280–3285 (2010).

20. Sobek, A. & Gustafsson, Ö. Latitudinal fractionation of polychlorinated biphenyls in surface seawater along a 62° N−89° N transect from the southern Norwegian Sea to the North Pole Area. *Environ. Sci. Technol.* **38**, 2746–2751 (2004).
