# Supplementary material for: Marine primary production enhances removal of hydrophobic organic contaminants from Eurasian Arctic shelf waters
Source: Nat Commun. 2026 Oct 23;17:11074. doi: 10.1038/s41467-026-78857-3 (PMC13694705; doi:10.1038/s41467-026-78857-3)
Supplement: Supplementary file 2 [file 41467_2026_78857_MOESM2_ESM.pdf]

The Description of Additional Supplementary Files

Title: Supplementary Data 1

Description:

Table S1. Sampling and biogeochemistry information and concentrations of polychlorinated biphenyls in sediment samples measured in the current study.

Table S2. Sampling and biogeochemistry information and concentrations of polychlorinated biphenyls in sediment samples in literature.

Table S5. Time series of export fluxes of marine organic carbon to sediment beds and the ratio of polychlorinated biphenyl sink associated with marine organic carbon between two scenarios.
