# Supplementary material for: Marine primary production enhances removal of hydrophobic organic contaminants from Eurasian Arctic shelf waters
Source: Nat Commun. 2026 Oct 23;17:11074. doi: 10.1038/s41467-026-78857-3 (PMC13694705; doi:10.1038/s41467-026-78857-3)
Supplement: Supplementary file 3 [file 41467_2026_78857_MOESM3_ESM.pdf]

nature portfolio

Peer Review File

Marine Primary Production Enhances Removal of Hydrophobic Organic Contaminants from Eurasian Arctic Shelf Waters

Corresponding Author: Dr Xiaodi Shi

This file contains all reviewer reports in order by version, followed by all author rebuttals in order by version.

Version 0:

Reviewer comments:

Reviewer #1

(Remarks to the Author)
Review of “Marine Primary Production Enhances Removal of Hydrophobic Organic Contaminants from Eurasian Arctic Shelf Waters”

This is a nice and interesting work on the controls of PCB accumulation in Arctic sediments and projections under a climate change scenario. The novelty arise at suggesting that plankton-derived organic matter is specially efficient transferring contaminants to the sediment dominating the contaminant fate. I only have some suggestions to improve the depth of the discussion aiming at clarifying the message.

- It is generally believed that marine OC is more aliphatic and terrestrial OC is more aromatic as noted by the authors. One would think that aromatic OC would have higher tendency for sorption of PCBs, but the observation is the opposite. I like the overall synthesis of this fact done in the introduction, which is built to great extent on author’s previous works. As there are works on PCB sorption in/on phytoplankton (Skoglund et al. 1996, Del Vento & Dachs 2002), I wonder if these have been taken into account in the synthesis. I would say that these predict phytoplankton-seawater partitioning close to Kow, while water-sediment partitioning is lower than Kow, by more than a factor of 2. What I suggest is that reviewing previous works with partitioning to cell cultures could support author’ claim.
- Then, maybe, the differences could arise from other factors. For example, it could be that marine OC is more labile, and as this OC degrades the PCB concentrations per gr of OC increase resulting in an apparent increased concentration. The role of preferential degradation organic matter in comparison to persistent contaminants amplifying the fugacity has been suggested before (reference 2, or Dachs et al. EST 2026 and references therein).
- Line 121. The magnitude of the decline is important, provide it in % or e-folding time.
- Line 122. Is the reduced transport because the water flow has decreased or because the concentration has decreased?
- Lines 125-133. Note that the differences in PCB congeners could also be related to biogeochemistry of primary production, in addition to primary sources. For PCB 28, the air-water is generally in equilibrium regardless the primary production, while for highly chlorinated PCBs (the more hydrophobic), organic matter becomes depleted in comparison to the atmospheric reference value as settling is faster than atmospheric deposition. This has been shown in other regions (Berrojalbiz et al. EST 2011, Morales et al. EST2015).
- Lines 155-157. It could be true. But it is not clear to me if the authors refer to concentrations or fluxes, and this distinction is important. Are the deposited fluxes equals, or are the concentrations equal?
- Line 187. It could be true as the relative flux of the more hydrophobic when compared with the water column reservoir is larger. The amplification of sediment concentrations due to degradation of labile OC could increase this for the marine influenced regions.
- Line 200. Again, a comparison of concentrations does not imply a comparison of fluxes (loadings).
- Line 202. The discussion of climate influence could start in a different paragraph….
- Line 235. Maybe use literature examples for PCBs not for PAHs.
- Despite reference 12, I have problems thinking that alkanes (aliphatic type) associate better with PCBs than PAH (aromatic). I think that part of the story is because the sorption originates from cells (high accumulation), and because aliphatic OC is more labile than aromatic OC.
- Figure 4 and associated text. Which is the uncertainty in the model estimates?

- Overall, this is a very nice work, but it only covers PCBs. It would be very interesting if other chemicals would have been added. For example PFAAs, which are also persistent, or other contaminants with lower persistence. Even if new databases cannot be incorporated, something could be said about persistence for other contaminants (higher primary production implies higher bacteria, and higher temperatures imply more degradation….).

Reviewer #2

(Remarks to the Author)
The manuscript appears well written and have used available data in a good manner. Several comparisons are done between Baltic Sea and Arctic. There is (often) a lack of data in the Arctic region, which means that some processes have to be investigated and referred to from other ecosystems. There is research conducted on e.g. land run-off to coastal areas and the role of particles for land-coast transport of POPs in a changing climate using Svalbard as case area. The present article do focus on organic carbon, which is obviously of high interest. But, if possible (given length restrictions etc.), a discussion on particles in general vs organic carbon in an Arctic perspective would have been interesting to see in the present article as well.

Change "hydrophobic organic contaminants (HOCs)" to "halogenated organic compounds" it is a more common established term, and cover the compounds mentioned in the article.

Table S4 is missing the the SI excel file.

Open Access This Peer Review File is licensed under a Creative Commons Attribution 4.0 International License, which permits use, sharing, adaptation, distribution and reproduction in any medium or format, as long as you give appropriate credit to the original author(s) and the source, provide a link to the Creative Commons license, and indicate if changes were made.
In cases where reviewers are anonymous, credit should be given to 'Anonymous Referee' and the source.
The images or other third party material in this Peer Review File are included in the article's Creative Commons license, unless indicated otherwise in a credit line to the material. If material is not included in the article's Creative Commons license and your intended use is not permitted by statutory regulation or exceeds the permitted use, you will need to obtain permission directly from the copyright holder.
To view a copy of this license, visit https://creativecommons.org/licenses/by/4.0/

Authors' responses to reviewer comments and resulting revisions to the manuscript "*Marine primary production enhances removal of hydrophobic organic contaminants from Eurasian Arctic shelf waters*" (Nature Communications, Manuscript Number NCOMMS-26-057973)

We sincerely thank the reviewers for their constructive feedback, which has clearly contributed to improve the manuscript. We are encouraged by the reviewers' overall positive remarks and their recognition of the significance of our study on high efficiency of marine organic matter (OM) to transfer hydrophobic organic contaminants (HOCs) to sediment in the Eurasian Arctic shelves. In particular, we have carefully addressed the comments concerning model uncertainties and influences of OM lability and OM settling flux on sediment contaminant concentrations. We have added discussions on both points.

Below, the reviewer comments are shown in black italic type, and for our point-by-point responses in black normal type. Our resulting revisions are in blue and are referred to line numbers in the final revised clean manuscript.

Reviewer #1 (Remarks to the Author):

Review of "Marine Primary Production Enhances Removal of Hydrophobic Organic Contaminants from Eurasian Arctic Shelf Waters"

This is a nice and interesting work on the controls of PCB accumulation in Arctic sediments and projections under a climate change scenario. The novelty arise at suggesting that plankton-derived organic matter is specially efficient transferring contaminants to the sediment dominating the contaminant fate. I only have some suggestions to improve the depth of the discussion aiming at clarifying the message.

- It is generally believed that marine OC is more aliphatic and terrestrial OC is more aromatic as noted by the authors. One would think that aromatic OC would have higher tendency for sorption of PCBs, but the observation is the opposite. I like the overall synthesis of this fact done in the introduction, which is built to great extent on author's previous works. As there are works on PCB sorption in/on phytoplankton (Skoglund et al. 1996, Del Vento & Dachs 2002), I wonder if these have been taken into account in the synthesis. I would say that these predict phytoplankton-

seawater partitioning close to Kow, while water-sediment partitioning is lower than Kow, by more than a factor of 2. What I suggest is that reviewing previous works with partitioning to cell cultures could support author' claim.

Response: This is a great point. Multiple laboratory studies reported fitted linear regression between log-transformed phytoplankton bioconcentration factors (BCFs) and log octanol/water partition coefficients (K_{ow}) for HOCs with log K_{ow} ranging from 4 to 6.5. (Koelmans, et al. *Environ. Toxicol. Chem.* 1999; Gerofke, et al. *Environ. Toxicol. Chem.* 2005; Fadaei, *Environ. Toxicol. Chem.* 2026). An examination of sorption data (mainly from soil) shows that the organic carbon/water partition coefficient (K_{OC}) is, on average, 0.35-fold of K_{ow} (Seth, et al., *Environ. Sci. Technol.* 1999). Using these fitted regression curves, we calculated that the predicted sorption potential of phytoplankton in culture experiments is about one to three times higher than that of soil OM for HOCs ($4 < \log K_{\mathrm{ow}} < 6.5$). We have added this discussion to the Introduction to support our hypothesis: "Existing data from sorption studies on phytoplankton and soil suggest that phytoplankton sorption potential is about three times higher than that of soil organic matter.[22-25]" (Lines 57-59)

"22. Koelmans, A. A., van der Woude, H., Hattink, J. & Niesten, D. J. M. Long‐term bioconcentration kinetics of hydrophobic chemicals in *Selenastrum capricornutum* and *Microcystis aeruginosa*. *Environ. Toxicol. Chem.* **18**, 1164–1172 (1999).

23. Gerofke, A., Kömp, P. & McLachlan, M. S. Bioconcentration of persistent organic pollutants in four species of marine phytoplankton. *Environ. Toxicol. Chem.* **24**, 2908–2917 (2005).

24. Fadaei, H., Place, A. & Ghosh, U. Improved estimates of PCB bioaccumulation factors for algae and zooplankton using passive sampling. *Environ. Toxicol. Chem.* **0**, 1–9 (2026).

25. Seth, R., Mackay, D. & Muncke, J. Estimating the organic carbon partition coefficient and its variability for hydrophobic chemicals. *Environ. Sci. Technol.* **33**, 2390–2394 (1999)." (Lines 588-599)

We have not included the two studies suggested by the reviewer (i.e. Skoglund, R. et al. *Environ. Sci. Technol.* 1996 and Del Vento, S. & Dachs, J. *Environ. Toxicol. Chem.* 2002), because both studies aimed to separate HOCs sorbed to the phytoplankton surface versus HOCs dissolved in the interior of the cells and the BCFs may thus not be comparable to our data.

- Then, maybe, the differences could arise from other factors. For example, it could be that marine OC is more labile, and as this OC degrades the PCB concentrations per gr of OC increase resulting in an apparent increased concentration. The role of preferential degradation organic matter in comparison to persistent contaminants amplifying the fugacity has been suggested before (reference 2, or Dachs et al. EST 2026 and references therein).

Response: We observed that the enhancement effect of marine OM on organic carbon (OC)-normalized concentrations of polychlorinated biphenyls (PCBs) in sediment increased with PCB hydrophobicity. Marine OM is broadly considered to be more labile than modern terrestrial OM (Burdige, D. J. *Chem. Rev.* 2007). The degradation of labile marine OM would increase PCB concentrations per gram of remaining OM. However, it remains unknown how the degradation of labile OM influences PCB congener distribution. For instance, vertical profiles of particle-phase PCB concentrations in the Mediterranean Sea showed no general trends of PCB congener distribution with depth (Dachs, J. et al. *Mar. Chem.* 1997). In the open Baltic Sea, the enrichment of PCBs from suspended particulate matter in surface water to the sediment was independent of hydrophobicity (Axelman, T. et al. *Ambio*, 2000). The Baltic Sea study also reported that K_{OC} of most PCB congeners between the dissolved phase and particulate organic carbon phase increased more than one order of magnitude from 12 m to 91 m depth, potentially due to the preferential degradation of proteins and carbohydrates relative to lipids (Axelman, T. et al. *Ambio*, 2000). Considering the potential increasing sorption potential of OM during vertical transport, it remains uncertain whether the degradation of labile OM would amplify the fugacity of PCBs in OM.

We therefore consider the degradation of labile OM as a factor contributing to the uncertainties of the relative importance of factors governing concentrations of PCBs and other contaminants in sediment OM of the Arctic Ocean (Figure 3 in the main text). We have added a discussion regarding the impacts of degradation of labile OM: "Additionally, this study uses sediment samples to infer sorption of HOCs to organic matter in the water column. Organic matter sorption and degradation processes in the water column can contribute to variability of HOC concentrations in the sediment. For instance, earlier field studies showed that sorption of HOCs to phytoplankton was negatively associated with biomass in the water phase,[57,58] and degradation of organic matter during vertical transport could amplify HOC concentrations per remaining OM.[59] Marine organic

matter is preferentially degraded relative to terrestrial organic matter during vertical transport.[13]" (Lines 250-257)

- Line 121. The magnitude of the decline is important, provide it in % or e-folding time.

Response: We have added the magnitude in this sentence: "Σ_7PCB concentrations within each shelf sea significantly declined by 50-60% between 1994 and 2020, in line with observed long-term declines in PCB air concentrations in the Arctic following their production phase-out.[41]" (Lines 117-119)

- Line 122. Is the reduced transport because the water flow has decreased or because the concentration has decreased?

Response: To improve clarity, we have revised this sentence: "Σ_7PCB concentrations within each shelf sea significantly declined by 50-60% between 1994 and 2020, in line with observed long-term declines in PCB air concentrations in the Arctic following their production phase-out.[41]" (Lines 117-119)

- Lines 125-133. Note that the differences in PCB congeners could also be related to biogeochemistry of primary production, in addition to primary sources. For PCB 28, the air-water is generally in equilibrium regardless the primary production, while for highly chlorinated PCBs (the more hydrophobic), organic matter becomes depleted in comparison to the atmospheric reference value as settling is faster than atmospheric deposition. This has been shown in other regions (Berrojalbiz et al. EST 2011, Morales et al. EST2015).

Response: We respond to this comment together with the one related to Line 187. Please see the response regarding Line 187.

- Lines 155-157. It could be true. But it is not clear to me if the authors refer to concentrations or fluxes, and this distinction is important. Are the deposited fluxes equals, or are the concentrations equal?

Response: We discuss sediment concentrations in this paragraph, which has been noted in the original version of the manuscript:

"Neither dry-weight nor OC-normalized PCB concentrations show significant decreases between 70° N and 80° N." (Lines 134-135)

"The stable, even increasing, latitudinal trends in sediment concentrations observed in this study therefore suggest that other factors decouple the PCB sink from declining northward PCB transport." (Lines 146-148)

"We therefore hypothesize that increasing f_{mar} could be one of the factors that offset the expected latitudinal decline in PCB transport, resulting in the spatially uniform PCB concentrations observed across the Eurasian Arctic shelf sediments." (Lines 152-154)

No changes were made in response to this comment.

- Line 187. It could be true as the relative flux of the more hydrophobic when compared with the water column reservoir is larger. The amplification of sediment concentrations due to degradation of labile OC could increase this for the marine influenced regions.

Response: We observed that the enhancement effect of marine OM on OC-normalized concentrations of PCBs in sediment increased with PCB hydrophobicity. The reviewer's suggested explanation for this observation requires the co-occurrence of low OM settling fluxes and high fraction of marine OM. As discussed in the second response to the reviewer, it remains unclear how the degradation of labile marine OM influences PCB congener distribution.

We calculated the mass accumulation rate of OC (MAR_{OC}; g cm^{-2} yr^{-1}), based on the mass accumulation rate and total organic carbon of the sediment (Tables S1 and 2 in Supporting Dataset). In the east (i.e., Laptev and East Siberian Seas), MAR_{OC} is not significantly associated with the fraction of marine OC (f_{mar}) in sediment (p = 0.09). In the west (i.e., Barents and Kara Seas), we established a bivariable regression model as below:

$$\lg C_{PCB} = a_1 \times \lg MAR_{OC} + a_2 \times f_{mar} + \varepsilon$$

where C_{PCB} is PCB sediment concentrations (ng g^{-1} OC) corrected for latitude and depositional year. The results are summarized in the table below.

Congener	a_1	*p*	a_2	*p*	R^2
PCB 28	-0.27	0.087	0.0059	<0.01	0.31
PCB 52	0.057	0.83	0.011	<0.01	0.2
PCB 101	-0.39	0.016	0.0062	<0.01	0.39
PCB 118	-0.23	0.22	0.0084	<0.01	0.33
PCB 138	-0.20	0.34	0.0090	<0.01	0.28
PCB 153	-0.29	0.12	0.0080	<0.01	0.35
PCB 180	-0.31	0.12	0.0074	<0.01	0.34

These results show that OC-normalized PCB sediment concentrations for all congeners, except PCB 101, are not significantly associated with MAR_{OC} in the west. The explanation proposed in this comment is therefore not applicable to sediment samples in this study. Two articles (Berrojalbiz, et al. *Environ. Sci. Technol.* 2011; Morales, et al. *Environ. Sci. Technol.* 2015) cited by the reviewer in the comment regarding Lines 125-133 studied phytoplankton samples and showed the negative correlation between biomass and HOC concentrations in phytoplankton. It therefore remains uncertain how accurately contaminant concentrations, MAR_{OC}, and f_{mar} derived for sediment samples can reflect those for particulate OM in the water column.

In the revised manuscript, we acknowledge that OM settling fluxes is also a factor contributing to the variability of the study (Figure 3 in the main text). We have added the following to the discussion regarding the variability from the OM settling flux: "Additionally, this study uses sediment samples to infer sorption of HOCs to organic matter in the water column. Organic matter sorption and degradation processes in the water column can contribute to variability of HOC concentrations in the sediment. For instance, earlier field studies showed that sorption of HOCs to phytoplankton was negatively associated with biomass in the water phase,[57,58] and degradation of organic matter during vertical transport could amplify HOC concentrations per remaining OM.[59] Marine organic matter is preferentially degraded relative to terrestrial organic matter during vertical transport.[13]" (Lines 250-257)

- Line 200. Again, a comparison of concentrations does not imply a comparison of fluxes (loadings).

Response: We have changed 'loadings' into 'concentrations' throughout the manuscript.

- Line 202. The discussion of climate influence could start in a different paragraph….

Response: We have started a new paragraph for this discussion.

- Line 235. Maybe use literature examples for PCBs not for PAHs.

Response: The reference number here was wrong and should be ref. 19. We aimed to study the factors influencing the fate of HOCs in ocean systems in general, not specifically PCBs. Indeed any HOCs, including polycyclic aromatic hydrocarbons, could be used as examples. Ref. 19 offers good support here, because it establishes the association between lipid carbon content and sorption potential using various organic matter precursors through sorption experiments. No changes were made in response to this comment.

- Despite reference 12, I have problems thinking that alkanes (aliphatic type) associate better with PCBs than PAH (aromatic). I think that part of the story is because the sorption originates from cells (high accumulation), and because aliphatic OC is more labile than aromatic OC.

Response: Phytoplankton cells contain lipids, which results in high accumulation of HOCs in phytoplankton. Reference 12 shows that the mobile amorphous sorption domain of lipids facilitates dissolution of HOCs into OM. Additionally, lipids are generally less polar than humic matter, the latter being a major component in soil. As discussed in the second response to the reviewer, lability of marine OM is considered as a factor contributing to the variability of the study (Figure 3 in the main text). Both points are therefore not included in this paragraph.

- Figure 4 and associated text. Which is the uncertainty in the model estimates?

Response: The model for estimating export fluxes of marine OC has previously been established, fine-tuned, and evaluated in Oziel, et al. *Nat. Clim. Chang.* 2025. There are three types of uncertainty for OC export fluxes, i.e., internal model uncertainty, scenario uncertainty, and inter model uncertainty. (1) Internal model uncertainty requires running the same simulation with different initial parameters hundreds of times and therefore cannot be estimated. (2) Scenario uncertainty is defined as and can be quantified by the difference between Shared Socioeconomic Pathway (SSP) 3-7.0 and 1-2.6. (3) Inter model uncertainty was evaluated through comparison with the Coupled Model Intercomparison Project Phase 6 (CMIP6) ensemble and has been shown in Oziel, et al. *Nat. Clim. Chang.* 2025. Briefly, comparison of OC export fluxes from the current model with 16 to 17 CMIP6 Earth System Models demonstrated consistent dynamics. Additionally, the modeled export flux was compared with the most comprehensive current observational dataset for the sinking of particulate OM. The modelled export flux was able to reproduce observations significantly and reasonably well with a noticeable improvement for the Arctic Ocean.

This study reported the ratio of sediment PCB sink associated with marine OC between SSP 3-7.0 and 1-2.6. To demonstrate changes in marine OC fluxes and in the associated capacity to remove HOCs from the water column in Eurasian Arctic shelves, we applied the fixed PCB concentrations per marine OC and temporal and latitudinal corrections for this calculation in both scenarios. The uncertainty of PCB sink ratios associated with marine OC therefore also lies in the estimation of marine OC export fluxes. We have added discussion regarding limitations and uncertainties in the Methods section:

"Please see Methods regarding limitations and uncertainties of these estimations." (Lines 272-273)

"The uncertainty of modeled export fluxes of marine OC has previously been evaluated in ref. 37. Briefly, scenario uncertainty can be quantified by the difference between SSP3-7.0 and SSP1-2.6. Inter model uncertainty was evaluated through comparison with the Coupled Model Intercomparison Project Phase 6 (CMIP6) ensemble. Comparison of OC export fluxes from the current model with those from 16 to 17 CMIP6 Earth System Models demonstrated consistent dynamics. The modeled OC export flux was also compared with the most comprehensive current observational dataset for the sinking of particulate organic matter. The modeled OC export flux was able to reproduce observations significantly and reasonably well with a noticeable improvement for the Arctic Ocean.[37]

To demonstrate changes in marine OC export fluxes and in the associated capacity to remove HOCs from the water column in Eurasian Arctic shelves, we applied the fixed PCB concentrations per marine OC and fixed temporal and latitudinal corrections for annual export fluxes of PCBs to sediment throughout the 21st century under both low- and high-emission scenarios. The uncertainty of PCB sink ratio associated with marine OC between two scenarios mainly lies in the modeled marine OC export fluxes. Since temperature is a strong factor influencing both volatilization and global fractionation of HOCs,[9] the fixed temporal and latitudinal corrections cannot fully reflect the influence of temperature change in the two modeled scenarios. Therefore, the current estimations cannot be interpreted as predictions of the magnitude of the PCB sink in Arctic shelf sediments." (Lines 488-509)

- Overall, this is a very nice work, but it only covers PCBs. It would be very interesting if other chemicals would have been added. For example PFAAs, which are also persistent, or other contaminants with lower persistence. Even if new databases cannot be incorporated, something could be said about persistence for other contaminants (higher primary production implies higher bacteria, and higher temperatures imply more degradation….).

Response: We use PCBs as one group of model HOCs, due to their wide range of hydrophobicity and extensive quantification in the Arctic. It is challenging to achieve our aim using other compounds with scarce measurements in the Arctic. The current results can be extrapolated to other compounds with comparable persistence and physical-chemical properties to PCBs. Additional content regarding persistence has been included in the revised manuscript:

"This climate-driven increase of the PCB sediment sink can be extrapolated to other HOCs with comparable persistence and physical-chemical properties." (Lines 292-294)

"Climate-driven changes in the Arctic may therefore alter the fate of HOCs not only through temperature-dependent physical processes (e.g., re-emission and partitioning) and (a)biotic degradation processes (e.g., bacterial degradation),[8,9] but also through reshaping the quantity and quality of organic matter that governs contaminant transport in the aqueous phase." (Lines 297-302)

Reviewer #2 (Remarks to the Author):

The manuscript appears well written and have used available data in a good manner. Several comparisons are done between Baltic Sea and Arctic. There is (often) a lack of data in the Arctic region, which means that some processes have to be investigated and referred to from other ecosystems. There is research conducted on e.g. land run-off to coastal areas and the role of particles for land-coast transport of POPs in a changing climate using Svalbard as case area. The present article do focus on organic carbon, which is obviously of high interest. But, if possible (given length restrictions etc.), a discussion on particles in general vs organic carbon in an Arctic perspective would have been interesting to see in the present article as well.

Response: Hydrophobic organic contaminants preferentially concentrate in OM through multimedia partitioning. OM, including OM in particles from coastal run-off, is therefore the most relevant matrix for the fate of hydrophobic organic contaminants. We have clarified this in the Introduction in the original version of the manuscript. No changes were made in response to this comment.

Change "hydrophobic organic contaminants (HOCs)" to "halogenated organic compounds" it is a more common established term, and cover the compounds mentioned in the article.

Response: We aim to study the factors influencing the fate of organic contaminants in ocean systems. The fate of organic contaminants depends on their physical-chemical properties, particularly hydrophobicity. Although halogenated organic contaminants cover the model compounds in the article, lots of halogenated contaminants are hydrophilic, such as perfluoroalkyl acids. Consequently, we assert that the term "halogenated organic compounds" is too broad for the current work. No changes were made in response to this comment.

Table S4 is missing the the SI excel file.

Response: Table S4 is in the original PDF file of Supplementary Information. No changes were made in response to this comment.
